# Extending Genetic Algorithms with Biological Life-Cycle Dynamics

**DOI:** 10.3390/biomimetics9080476

**Published:** 2024-08-06

**Authors:** J. C. Felix-Saul, Mario García-Valdez, Juan J. Merelo Guervós, Oscar Castillo

**Affiliations:** 1 Division of Graduate Studies and Research, Tijuana Institute of Technology, Tecnológico Nacional de México (TecNM), Tijuana 22414, Mexico; d20210001@tijuana.tecnm.mx (J.C.F.-S.); ocastillo@tectijuana.mx (O.C.); 2 Department of Computer Engineering, Automatics and Robotics, University of Granada, 18071 Granada, Spain; jmerelo@ugr.es

**Keywords:** evolutionary algorithms, genetic algorithms, computational optimization, bio-inspired algorithms

## Abstract

In this paper, we aim to enhance genetic algorithms (GAs) by integrating a dynamic model based on biological life cycles. This study addresses the challenge of maintaining diversity and adaptability in GAs by incorporating stages of birth, growth, reproduction, and death into the algorithm’s framework. We consider an asynchronous execution of life cycle stages to individuals in the population, ensuring a steady-state evolution that preserves high-quality solutions while maintaining diversity. Experimental results demonstrate that the proposed extension outperforms traditional GAs and is as good or better than other well-known and well established algorithms like PSO and EvoSpace in various benchmark problems, particularly regarding convergence speed and solution qu/ality. The study concludes that incorporating biological life-cycle dynamics into GAs enhances their robustness and efficiency, offering a promising direction for future research in evolutionary computation.

## 1. Introduction

Biological processes have always inspired evolutionary algorithms, embodying the principle *“Let nature be your guide”* [1]. This bio-inspired approach spans various research fields, including artificial intelligence, machine learning, and complex system modeling.

In particular, evolutionary algorithms (EAs) have been successfully applied to solving complex optimization problems by mimicking the adaptive processes observed in the natural world [2]. Genetic Algorithms (GAs) [3,4] are canonical examples of these adaptive algorithms, using a population of candidate solutions and a process inspired by natural selection to improve the proposed solutions. These algorithms are generally designed to find optimal or near-optimal solutions by iteratively applying an evolutionary process to the population of candidate solutions. The process consists of these tasks: creating an initial population of potential solutions to the optimization problem (initialization), selection, the interchange of genetic material (through crossover or other operators), random change (through mutation or other mechanism), and termination criteria. Many other optimization meta-heuristics inspired by natural processes have been proposed [5,6,7,8,9], but in this work, we are interested in algorithms that operate on populations of candidate solutions. Some noteworthy examples are the Particle Swarm Optimization (PSO) [10,11] algorithm that simulates the social behavior of birds and the Ant Colony Optimization algorithm, inspired by the foraging behavior of ants [12,13].

A critical challenge of these algorithms is that they can incur high computational cost, mainly because evaluating the fitness of each candidate solution can be computationally expensive, especially for difficult optimization problems where each evaluation involves solving complex equations or running time-consuming simulations. Researchers are actively exploring various strategies to mitigate these costs and reduce execution times. A common approach is using parallel and distributed computing to significantly reduce the time required to find optimal solutions by executing multiple evaluations simultaneously across multiple processors or computing nodes. Some versions of these algorithms have been adapted to work in parallel by distributing the processing using many smaller populations [14,15,16] or multiple threads or nodes [17,18,19,20] to evaluate the fitness of candidate solutions. This multi-population approach mimics the concept of island models [21], where each population evolves independently.

In early studies [22,23], we proposed using cloud computing to reduce the execution time of evolutionary algorithms by using an event-driven architecture for the asynchronous execution of isolated populations. This architecture is independent of the underlying population-based algorithm because it is applied at the population level. In these works, populations are data units that are asynchronously processed by processing nodes that communicate with each other through a message broker.

An advantage of having multiple isolated populations is that they can maintain diverse solutions across different nodes, reducing the risk of premature convergence to local optima. These algorithms can combine local search strategies with global communication, leading to a better balance between exploration and exploitation. Asynchronous algorithms do not require all processes to wait during communication, which can lead to reduced waiting times and better use of computational resources.

In this paper, we propose an algorithm that keeps the advantages of the asynchronous, event-driven, multi-population approach we just mentioned but uses a single population instead. The algorithm is an extension to the canonical GA but adds to the model the parallel and asynchronous nature of the real world.

The algorithm applies the operators found in a GA through the following life cycle stages: growth, reproduction, and death. These stages can be applied in parallel to individuals in a population following the main characteristics of a steady-state genetic algorithm (SSGA) [24,25]. Unlike traditional GAs, which replace the entire population at each generation, SSGAs update the population continuously [26]. This involves selecting a few individuals, creating offspring, and then replacing some individuals in the population, typically one or two, in each iteration. By replacing only a small portion of the population at a time, SSGAs reduce the disruption caused by large-scale replacements seen in generational GAs. This strategy helps preserve good solutions and maintain diversity, by keeping in the population different individuals in different areas of the search space. In our proposal, individuals have an age attribute and the GA mechanisms for parent selection, mutation, and replacement of individuals are applied asynchronously to them within the population, considering that each individual is in a particular life cycle stage.

The main contributions of this paper are as follows:

- We propose the Life Cycle Genetic Algorithm (LCGA), an extension of an SSGA, that incorporates the life cycle stages of animal species with an asynchronous execution model that allows individuals to have an independent life cycle.
- We incorporate the concept of age into the individuals in the population, which controls the application of genetic operators and the removal of individuals.
- In the event of population extinction, we also introduce a population restart mechanism based on the best historical solutions.

The experiments section of our paper comprehensively evaluates LCGA’s performance. The first evaluation uses the COCO framework’s evaluation data to compare LCGA against the GA, PSO, and EvoSpace algorithms. For our final experiment analysis, we used the CEC-2017 mathematical functions to compare the LCGA against the Fuzzy Marine Predator Algorithm (FMPA) [27], a recent nature-inspired algorithm with similar characteristics.

The paper’s organization or structure is as follows. Section 2 shows a literature review and discusses existing genetic and evolutionary algorithms, identifying gaps that LCGA aims to fill. Section 3 focuses on our proposed algorithm description and details of the stages of the algorithm—birth, growth, reproduction, and death—drawing parallels to biological processes. Section 4 shows the experimental setup and results; it presents the methodology for testing LCGA’s efficacy against benchmark problems and other algorithms, followed by Section 5, a discussion. This section analyzes the obtained results. Finally, Section 6 focuses on the conclusion and future work, summarizes findings, highlights LCGA’s contributions to evolutionary computation, and suggests directions for further research.

## 2. Related Work

We now present a brief overview of current population-based optimization algorithms and lifecycle models related to our work, focusing on the main concepts and strategies they employ to solve complex optimization problems. Building upon the foundation laid by Holland [3], other EAs have been proposed.

An important EA variant is called Evolutionary Strategies (ES) [28], a type of algorithm that focuses on optimizing real-valued functions, which uses mutation as the main operator with recombination, when used, combining the parameters of parents to generate the offspring. In ES, several replacement strategies consider the offspring and the parents when selecting individuals to continue in the next population. A successful type of ES is the Covariance Matrix Adaptation Evolution Strategy (CMA-ES) [29]; this algorithm is particularly effective in high-dimensional and complex problem spaces [30]. CMA-ES iteratively generates improved candidate solutions by sampling from a multivariate normal distribution centered around a mean vector, which is updated based on the selected top-performing individuals. The algorithm dynamically adapts the covariance matrix, capturing variable dependencies and adjusts the step size to balance exploration and exploitation. In contrast with a traditional GA, in CMA-ES, properties of the population as a whole are used to generate new siblings. Later successful variants of this algorithm include RB-IPOP-CMA-ES [31].

The approach applied by ES is similar to the one in Estimation of Distribution Algorithms (EDAs) [32], where a selected set of solutions in the population is used to estimate a probability distribution that generates new candidate solutions. This probability distribution depends on analyzing frequencies of the most successful individuals, the elite. In this paper, we will employ a similar strategy in the context of restarting the algorithm once the population has been extinguished.

Other properties of the population are used by other evolutionary algorithms, for instance, Differential Evolution (DE) [33] considers distance information in the population to generate new candidate solutions that are random deviations of current solutions. This algorithm has also evolved and spawned many variants that have reached very competitive results in the COCO benchmarks, which we are using in this paper; variants such as JADE [34], SHADE  [35], or SaDE [36].

In DE, new candidate solutions are generated by adding the weighted difference between two randomly selected parents. Candidate solutions are then evaluated against predetermined individuals, and if they have better fitness, they replace the individual with which they were compared; otherwise, they are discarded. On the other hand, other bio-inspired algorithms, such as the Particle Swarm Optimization (PSO) [10,11], also consider the information of the population but go even further by considering the position of each candidate solution in the search space. Unlike GAs that involve genetic operations and selection processes, PSO simulates a continuous flow of particles across the search landscape. Crucially, particles are not replaced between iterations but can adjust their positions based on interactions and shared information within the swarm, allowing them to identify local or global optima effectively. The PSO algorithm has inspired the evolutionary counterpart; an example is the neighborhood-based mutation operator [37]. This operator uses a neighborhood structure to guide mutation, enhance local search capability, and balance exploration and exploitation. It is similar to the mutation operator found in a GA but focuses on exploiting local information, which is considered more efficient in specific scenarios. Other Swarm Intelligence (SI) algorithms add some form of hierarchy to the population; for instance, the Gray Wolf Optimizer (GWO) [38] divides the population into a hierarchy that intends to balance exploration and exploitation effectively. We consider the techniques found in these algorithms to be relevant to the design of EA variants.

More recent bioinspired algorithms use some form of adaptation of parameters. An instance of these algorithms is the Fuzzy Marine Predator Algorithm (FMPA), which integrates a generalized type-2 fuzzy system with the Marine Predator Algorithm (MPA) [39] to dynamically adjust parameters and enhance optimization performance. This combination allows FMPA to effectively handle uncertainties and balance exploration and exploitation through the predator-prey model. FMPA has demonstrated superior results on CEC-2017 benchmark functions and is particularly effective in optimizing fuzzy controllers for mobile robots. Its robust performance, adaptability, and advanced handling of uncertainties make it a worthy benchmark for comparing and evaluating our research results for developing and improving the LCGA algorithm.

We also follow the research of Salgotra et al. [40], which introduced an enhanced version of the Cuckoo Search (CS) algorithm, termed CSsin, which incorporates several modifications to improve its performance on the CEC 2017 and CEC 2020 benchmark problems. Some of its key enhancements include new global and local search techniques, a dual search strategy to balance exploration and exploitation, a linearly decreasing switch probability to maintain this balance, and a linearly decreasing population size to reduce computational load. We also compare our algorithm against this enhanced version of the CS algorithm.

Some works explore the life cycle of organisms to improve the optimization process. An early example is the LifeCycle Model proposed by Krink et al. [41] designing a hybrid optimization framework inspired by biological life cycles, a model that integrates three distinct optimization techniques: Particle Swarm Optimization (PSO), genetic algorithms (GA), and hill climbers (HC). This model allows each candidate solution to dynamically transition between these three optimization methods based on performance and fitness improvements. The LifeCycle model capitalizes on the strengths of each technique: PSO for exploration, GA for diversification and recombination, and HC for fine-tuning local solutions. This self-adaptive strategy balances exploration and exploitation, aiming to improve overall optimization performance. Both the LifeCycle Model and our research share a foundation in biological inspiration and self-adaptation, enhancing the optimization processes through hybrid approaches. While the LifeCycle Model employs distinct transitions between PSO, GA, and HC, our research focuses on evolutionary dynamics within genetic algorithms, potentially offering more subtle transitions within a single evolutionary framework.

In another paper, Karami et al. [42] presented an evolutionary optimization algorithm inspired by the natural life cycle of plants, the Plant Life Cycle (PLC) algorithm. It is based on biological processes such as pollination, fertilization, seed dispersal, and local competition to find optimal solutions. Compared to our research, both algorithms share a foundation in biological inspiration and structured optimization processes. However, the PLC algorithm uniquely integrates plant-inspired strategies, while our study focuses on evolutionary dynamics within the genetic algorithm framework, aiming for advanced evolutionary strategies and efficient optimization in diverse and complex problem spaces.

Finally, Zhong et al. [43] introduced two novel strategies to enhance the Vegetation Evolution (VEGE) algorithm. The dynamic maturity strategy allocates more resources to better-performing individuals, allowing them to generate more seeds based on their fitness, promoting competition, and improving search efficiency. Both the enhanced VEGE algorithm and our research draw inspiration from natural processes to improve optimization performance. They incorporate adaptive strategies to dynamically enhance their ability to find global optima, and emphasize maintaining population diversity to prevent premature convergence. However, VEGE uses a plant-inspired strategy focusing on growth and seed dispersal phases, while our research focuses on evolutionary dynamics within genetic algorithms. VEGE employs diverse mutation methods to enhance diversity, whereas our approach uses different mutation and crossover techniques.

A concept we are using in this algorithm, age, has also inspired different algorithms, mainly in the context of another bio-inspired algorithm, artificial immune systems, although it had been previously introduced in the realm of genetic algorithms [44]. This algorithm assigned an age to every individual, as well as a maximum age. This changes every generation, with mutation and crossover rates depending on the age. The “survival rate” of an individual depends not only on fitness, but also on age, with probability descending up to a maximum age, and birth rate following a “lifecycle” pattern, increasing up to an “adult” age, then decreasing. The use of age-stratified population is analyzed theoretically, and its results were proved successful empirically. A similar approach, called the age-layered population structure (ALPS), was introduced slightly later [45]. This algorithm introduces diversity by the random inclusion of 0-aged individuals, but organizes the population by strata according to their age, which is related to the age of their oldest parent (we should maybe note that using here the concept of age is maybe stretching the metaphor a bit too far). By restricting reproduction and other operators to individuals within the same age stratus, they avoid domination of the population by “older” individuals with a higher fitness, allowing the exploration of different areas of the fitness landscape. The introduction of random individuals is similar to the restart step that we introduce in this algorithm.

In our case, we propose an extension to the original GA, adding some of the elements described above but keeping the main structure of the GA. In our previous work, we proposed a distributed EA cloud-native architecture  [23] to run multiple EA instances in parallel, including GAs, PSO, and other population-based variants. Mixing distinct EAs with different characteristics and behaviors in a single run gave favorable results, showing the potential of combining different algorithms. Expanding this idea, we intended to create a new EA that could asynchronously parallelize various operators and strategies using a single population and, if possible, a single metaphor. The algorithm mimics the life cycle of animals, which naturally incorporates some of the elements found in the above-related works.

## 3. Algorithm Proposal

**Figure 1:**
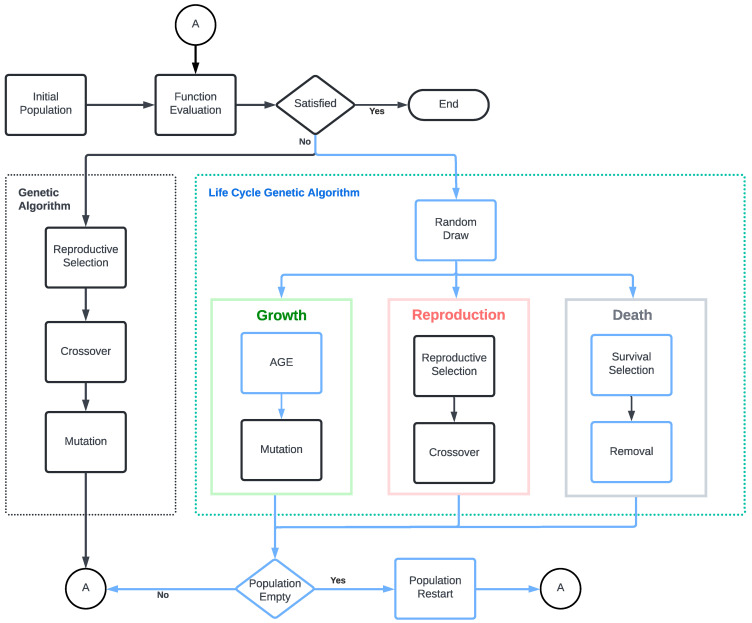
General model concept for the LCGA algorithm.

We show the general concept of the proposed algorithm in contrast with a standard GA in Figure 1. To the left, we have a GA where the genetic operators are executed sequentially and applied to the entire population. After each iteration, a new generation replaces the original population. To the right, we have the LCGA algorithm. In this case, operators are not applied sequentially to the entire population but to samples of individuals. This means that different GA operators are applied to individual population members simultaneously. Operators are distributed as independent processes, each representing a life cycle stage. The parallel execution of each stage mimics the asynchronous and continuous population dynamics observed in biological systems. Stages are represented by boxes of different colors, showing the operators being executed as flowchart boxes. Standard GA operators are displayed in black, while LCGA operators and flow are shown in light blue. Stages do not have a sequence and can be executed in parallel.

The algorithm does not have generations; instead, it uses an age parameter to control the size of the population by removing unwanted individuals and replacing them with new ones. The growth stage increases the age of individuals and applies the mutation operator. The reproduction stage is responsible for selecting parents and applying the crossover operator. Finally, the death process selects the oldest, unfit individuals in the population and removes them. When required, the algorithm restarts the population with the best historical solutions if the population becomes extinct.

As a proof of concept, in this paper, we decided to execute the processes stochastically to simulate these characteristics. After a population sample is taken, a stage is chosen randomly and applied to the sample; this is shown in Algorithm 1. These processes could be executed in parallel in a distributed environment, allowing for a more efficient and dynamic execution.

The algorithm requires the following parameters: *P* is a reference to the empty population, PS is the population size, *D* is the dimensionality of the problem, LB is the lower bound of the search space, and UB is the upper bound. The algorithm also requires a reference to fitness function to to be used in the evaluation process Fitness. As mentioned earlier, the current algorithm selects a random stage to execute.

As mentioned, our algorithm follows the classic genetic algorithm, where all the individuals (candidate solutions) have a genotype (or chromosome) composed of a list of values. We calculate their fitness with the evaluation of the Fitness function. Next, we explain each of the stages in detail.
**Algorithm 1** LCGA main pseudocode**Require:** 
P=PS,D,LB,UB,Fitness InitializePopulation(P, PS, D, LB, UB, Fitness)   Evaluate(P, Fitness)1:**while** solution not found and termination conditions not met **do**2:    stage← RandomStage(1, 3)3:    **if** stage=1 **then**4:        GrowthStage5:    **else if** stage=2 **then**6:        ReproductionStage7:    **else**8:        DeathStage9:    **end if**10:    **if** P= **then**11:        PopulationRestart12:    **end if**13:    Evaluate(P, Fitness)14:**end while**

### 3.1. Population Initialization

The first steps of the algorithm are the same as in a traditional GA, where we initialize the population with a set of randomly created individuals. When initializing the population, diversity is essential to the evolutionary processes [46], which is why, in the Population Initialization phase, we generate new random individuals to join the initial population. The initialization procedure is shown in Algorithm 2, which requires the following parameters: *P* is a reference to the empty population, PS is the population size, which is number of individuals to be generated by the procedure, *D* is the dimensionality of the problem, LB is the lower bound of the search space, and UB is the upper bound. We use the GenerateChromosome procedure to create a new random individual, and then we add it to the population.
**Algorithm 2** Initialize Population Procedure1:**procedure** InitializePopulation(P, PS, D, LB, UB)2:    **for** n=1,…,PS **do**3:        chromosome← GenerateChromosome(D, LB, UB)4:        AddToEvaluation(P, chromosome)5:    **end for**6:**end procedure**

### 3.2. Evaluation

Following initialization, the algorithm evaluates each individual’s fitness in the same manner as a GA, where the genotype undergoes evaluation to determine its aptitude.

As we can see in Algorithm 3, the evaluation process is also responsible for controlling and verifying whether the exit conditions require terminating the experiment, such as whether it has found an acceptable solution or reached the maximum number of evaluations. Evaluation must be performed after each individual’s life cycle stage. The algorithm requires a reference to fitness function to to be used in the evaluation process Fitness and the population *P* to be evaluated. The algorithm keeps track of the number of evaluations performed, and if the termination conditions are met, the experiment is terminated.
**Algorithm 3** Evaluation1:**procedure** Evaluate(P, Fitness)2:    **for all** individuals in P **do**3:        **if** individual is not evaluated **then**4:           new_fitness← Fitness(individual)5:           UpdateIndividual(individual, new_fitness)6:           IncrementEvaluations7:        **end if**8:        **if** TerminationConditionsMet **then**9:           TerminateExperiment10:        **end if**11:        AddToPopulation(P, individual)12:    **end for**13:**end procedure**

### 3.3. Growth

The growth process will take sample of individual to increase their age parameter, and apply with certain probability the mutation operator. We display the growth stage in Algorithm 4. The algorithm requires a reference to the population *P* to take the sample to be grown, and the sample size SS to be used in the growth process. For each individual in the sample, we increment their age and calculate the mutation rate.
**Algorithm 4** Growth1:**procedure** GrowthStage(P)2:    sample← GetPopulationSample(P, SS)3:    **for all** individuals in sample **do**4:        IncrementAge(individual)5:        mutation_rate← CalculateMutationRate(individual)6:        **if** PerformMutation(individual, mutation_rate) **then**7:           AddToEvaluation(individual)8:        **else**9:           AddToPopulation(individual)10:        **end if**11:    **end for**12:**end procedure**

LCGA introduces a dynamic mutation rate based on the specific age of each individual. We propose using a cosine function scaled to age. This ratio resembles adapting the mutation rate parameter values at runtime [47]. We chose the cosine function because it shows a curve similar to the growth of an individual. Age is scaled to π (π is equivalent to the maximum age in years) to evaluate age in scale with the cosine function. We convert it to a percentage to calculate the absolute value and round it to integers. When the mutation rate exceeds the maximum, we adjust it according to the limit configured as “max mutation”.

(1) $$age_{sc}=\pi \left(\frac{age}{age_{max}}\right)$$

(2) $$\mu =|100\cdot \cos(age_{sc})|$$

where π is the ratio between the circumference of a circle and its diameter, with a value of approximately 3.14159, $age_{sc}$ is the ratio of age on a π scale, with values between 0 and π, age is the age of the individual, $age_{max}$ is the maximum age to continue in the population, and μ is the mutation rate.

### 3.4. Reproduction

The reproduction stage, shown in Algorithm 5, starts with selecting two parents from population *P* by performing a tournament selection from a random population sample of size *k*. Once we have the two parents, we initiate the crossover process. We propose an alternative strategy that uses the genetic information from the parents to determine the range of values from which the offspring is going to be created. In this strategy, the value of each corresponding gene in the two parents is used as the lower and upper limits of a range. A new value for the corresponding gene in the offspring is then randomly selected from within this range. This is equivalent to a BLX-α crossover with α=0 [48] or a restricted intermediate recombination [49]. Mathematically, this can be expressed as follows.

Let us denote the two parents as $P_{1}$ and $P_{2}$, and let their corresponding genes be $g_{1}$ and $g_{2}$, respectively. For a given gene position *i*:

- The gene in the first parent at position *i* is $g_{1}^{i}$.
- The gene in the second parent at position *i* is $g_{2}^{i}$.

**Algorithm 5** Reproduction1:**procedure** ReproductionStage(P, k)2:    sample← GetPopulationSample(P, k)3:    parent1← SelectParentTournament(S)4:    **if** parent1 is not null **then**5:        sample← GetPopulationSample(P, k)6:        parent2← SelectParentTournament(S)7:        **if** parent2 is not null **then**8:           offspring← PerformCrossover(parent1, parent2)9:           **for all** child in offspring **do**10:               AddToEvaluation(child)11:           **end for**12:           AddToPopulation(P, parent2)13:        **end if**14:        AddToPopulation(P, parent1)15:    **end if**16:**end procedure**

In this strategy, the new value for the corresponding gene in the offspring at position *i*, denoted as $g_{\mathrm{offspring}}^{i}$, is randomly selected from a range determined by $g_{1}^{i}$ and $g_{2}^{i}$. Mathematically, this can be expressed as:

$$g_{\mathrm{offspring}}^{i}∼\mathrm{Uniform}\left(\min\left(g_{1}^{i},g_{2}^{i}\right),\max\left(g_{1}^{i},g_{2}^{i}\right)\right)$$

where Uniform(a,b) represents a uniform distribution between *a* and *b*. This means that $g_{\mathrm{offspring}}^{i}$ is a random value drawn from the interval $\left[\min\left(g_{1}^{i},g_{2}^{i}\right),\max\left(g_{1}^{i},g_{2}^{i}\right)\right]$.

In summary, for each gene position *i*, the offspring’s gene $g_{\mathrm{offspring}}^{i}$ is randomly chosen within the range defined by the corresponding genes in the parents, ensuring that the new gene value lies between the values of the two parents’ genes.

Following the algorithm, we add the offspring to the evaluation queue to calculate its fitness. The evaluation queue is a list of individuals that need to be evaluated, and it is processed by the evaluation procedure. We also add the parents back to the population.

### 3.5. Death

To maintain population balance and impose survival pressure, we use the death stage. We use an incremental rate that represents the adversities of nature, seen as the environmental demands required for the survival of individuals.

As shown in Algorithm 6, the death stage requires a reference to the population *P* to be evaluated. We take a sample of the population to evaluate each individual. We calculate the relative fitness of each individual in the sample, and compare it with the dynamically adjusted survival threshold. If the individual’s relative fitness is not greater than the survival threshold, the individual is removed from the population. The survival threshold is calculated using Equation (3), which adjusts the threshold value based on the number of times the death process has been executed and the maximum number of evaluations. The algorithm starts with a minimum value $survive_{min}$ and progresses to a maximum value $survive_{max}$, increasing the selection pressure. The pressure factor is used to reduce the steps needed to reach $survive_{min}$, where pressure≥1 and pressure<evalmax.
**Algorithm 6** Death1:**procedure** DeathStage(P)2:    sample← GetPopulationSample(P, sample_size)3:    **for all** individuals in sample **do**4:        **if** $individual_{age}<age_{max}$ **then**5:           **if** $individual_{fitness}>fitness_{best}$ **then**6:               $fitness_{best}\leftarrow individual_{fitness}$7:               AddToPopulation(P, individual)8:           **else**9:               $fitness_{relative}\leftarrow fitness_{best}/individual_{fitness}$10:               **if** SurvivalThreshold($fitness_{relative}$) **then**11:                   AddToPopulation(P, individual)12:               **end if**13:           **end if**14:        **end if**15:    **end for**16:**end procedure**

#### Survival Threshold (survival)

To decide if an individual survives or dies, we consider the following elements:

- Age: If an individual is older than a maximum age, the individual dies and is removed from the population. Fitness is ignored in this case.
- Best fitness: If the individual is young enough and has the best fitness in the population, it survives.
- Relative fitness: If the relative fitness of the individual is greater than the dynamically adjusted survival threshold, the individual survives.

The relative fitness of an individual is established by comparing its fitness with the best fitness found in the population. This is only evaluated for those individuals that do not have the best fitness. The fitness is expressed as a ratio between the individual’s fitness $fitness(ind_{i})$ and the best fitness best found in the population. In the case of a minimization problem, the ratio is calculated as $best/fitness(ind_{i})$, and because $fitness(ind_{i})>best>0$, the domain is [0,1], we multiply this value by 100 to have a domain of [0, 100].

The relative fitness is compared against a survival threshold survival that is dynamically calculated. The threshold value starts with a minimum value $survive_{min}$ to a maximum value $survive_{max}$, increasing as the algorithm progresses. If the current relative fitness of the individual is not greater than the survival threshold, the individual dies and is removed from the population. The threshold starts with a low value to allow fitness that is not too close to the best, and progresses to a higher value to increase the selection pressure. To keep track of the progress, we use the number of times the death process has been executed $death_{i}$ and the maximum number of evaluations evalmax established for the algorithm (evalmax>0). We can use a pressure factor to reduce the steps needed to reach $survive_{min}$, pressure≥1, and pressure<evalmax.

Below, we show Equation (3), required to calculate this value:

(3) $$survival=survive_{min}+death_{i}\cdot \frac{survive_{max}-survive_{min}}{evalmax}\cdot pressure$$

where $death_{i}$ is the number of process interactions with the population, $survive_{min}$ is the starting-point initial lower value to allow fitnesses not too close to the best, $survive_{max}$ is the final target, a higher value to increase the selection pressure progressively, evalmax is the maximum number of evaluations to interrupt the experiment, pressure is a multiplier to the increment in the survival threshold, and survival is the dynamically adjusted survival threshold required at this time.

In our initial test experiments, we used a survival threshold ($survive_{min}$ and $survive_{max}$, respectively) of 80 to 100 with pressure=1, and at the end of the evaluations, found that when we operated these values, a population with great diversity is generally far from our expected solution and not a satisfactory result. To achieve a more solid convergence, we decided to increase the pressure to 3.5, and saw a better performance on the algorithm behavior where it slightly exceeded the results of a GA. Following this trend, we decided to increase the pressure to 6 for all the experiments presented in this research, with which obtained the best outcomes, as shown in the experiments Section 4 of this document.

### 3.6. Restart

As mentioned, the main task of the death stage is to add selective pressure to the population. A consequence of this is that as time progresses, all individuals in the population can eventually be removed. Other authors have used similar strategies to remove specific individuals from the population or even delete the entire population if stagnation is detected. For instance, in Guimaraes et al. [50], an annihilator operator is proposed to delete duplicate individuals and those with a domain-specific range of values. The annihilator operator can also be applied to remove the entire population if the population converges to a particular minimum.

**Figure 2:**
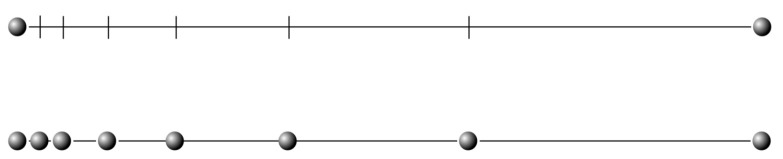
Fibonacci-based projection between two scalars; the lines are derived from the golden ratio.

**Figure 3:**
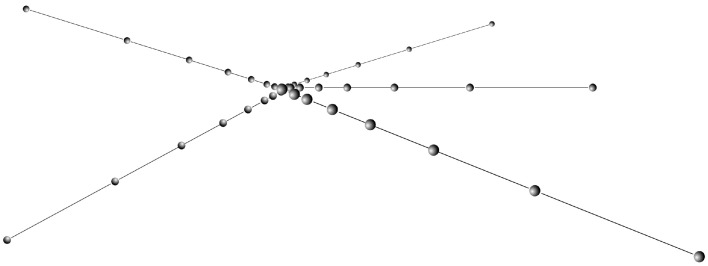
Fibonacci-based generation of new points in 3D space.

To restart a population, we propose the generation of new individuals that are in the vicinity (in the search space) of the best individual. To achieve this, we use the best individual found so far, the champion, and a another member of the elite collection, chosen at random. We generate new individuals between these two points, but not in a linear way, but using the Fibonacci sequence. We call this strategy a Fibonacci projection, where in this context, projection refers to the generation of new intermediate points between two points, by using a rule or transformation, in this case, distances (velocity) derived from the Fibonacci sequence, using the golden ratio [51,52,53,54,55]. For instance, if we have two scalars *a* and *b*, we can generate new points between them by following a Fibonacci sequence, as shown in Figure 2. By selecting other points, and now showing the results in 3D space, we can generate the points shown in Figure 3. We briefly mention this strategy in a previous work [56].

We detail the restart mechanism in Algorithms 7 and 8, but first, we explain the main variables and structures used:

- Let *E* be an ordered list (by fitness) of the elite individuals;
- Let $E_{\mathrm{best}}$ be the best individual (E[0]) and $C_{\mathrm{best}}$ be its chromosome;
- Let *P* be the previous population;
- Let PS be the size of the original population;
- Let $P_{\mathrm{new}}$ be the new population being created;
- Let $E_{\mathrm{random}}$ be a randomly selected elite individual and $C_{\mathrm{random}}$ be its chromosome;
- Let GetFibonacciChromosome be the function that modifies the chromosome using a Fibonacci-based method;
- Let + be an operator that sums two chromosomes as vectors;
- Let FM the number of individuals to generate in each iteration.

**Algorithm 7** Restart (Fibonacci projection)1:**function** CreateFibonacciPopulation($P,E,E_{\mathrm{best}},PS,FM$)2:    **if** P=[] **then**3:        $P_{\mathrm{new}}\leftarrow \left\{E_{\mathrm{best}}\right\}$4:        **while** $|P_{\mathrm{new}}|<PS$ **do**5:           $E_{random}∼\mathrm{Uniform}\left(E\right)$.6:           $P_{\mathrm{new}}^{\prime }=P_{\mathrm{new}}\cup \left\{E_{random}\right\}$.7:           x←08:           **while** x<FM **do**9:               $C_{\mathrm{random}}\leftarrow$GetFibonacciChromosome($C_{\mathrm{random}}$)10:               $C_{\mathrm{new}}\leftarrow C_{\mathrm{random}}+C_{\mathrm{best}}$                                                                ▹ Vector addition11:               $P_{\mathrm{new}}^{\prime }\leftarrow P_{\mathrm{new}}\cup \left\{C_{\mathrm{new}}\right\}$12:               **if** $|P_{\mathrm{new}}|\geq PS$ **then**13:                   **break**14:               **end if**15:               x←x+116:           **end while**17:        **end while**18:        **return** $P_{\mathrm{new}}$19:    **end if**20:**end function**

**Algorithm 8** Get Fibonacci Chromosome
1:**function** GetFibonacciChromosome(C)2:    x←03:    d← Length(chromosome)4:    F←[]5:    goldenRatio ← 1.618033988756:    goldenRatio ← goldenRatio × Uniform(0.95, 1.05) ▹ Randomize the golden ratio7:    **while** x<d **do**8:        $F_{x}\leftarrow \frac{C_{x}}{goldenRatio}$9:        x←x+110:    **end while**11:    **return** *F*12:
**end function**



### 3.7. Complexity of the Optimization Method

The computational complexity of the algorithm described earlier, like most population-based algorithms, is primarily influenced by the evaluation of the fitness functions. Therefore, a more accurate estimation of performance is the number of function evaluations needed to reach a particular target. The BBOB benchmark functions provide reports on this estimated performance. Assuming the fitness function operates as a black box with a complexity of O(1) and is executed sequentially, the overall complexity of the algorithm can be described as O(m·n), where *m* is the number of iterations and *n* is the population size. Initialization of the population has a complexity of O(n), with *n* representing the number of candidate solutions. After evaluation, there is a step for updating the solution, which typically has a complexity of O(m·n)+O(m·n·l), where *l* is the number of parameters in the fitness function. Finally, sorting the population by fitness adds a complexity of O(nlogn).

Regarding space complexity, the algorithm requires O(n) space to store the population and the historically best-found individuals used for population restart.

## 4. Experiments

The experimental evaluation of LCGA involves comparing our algorithm with the referenced single objective, continuous mathematical functions included in the COCO Benchmark Framework [57], to compare LCGA against other classical bioinspired algorithms, such as GA and PSO, a distributed version of GA and PSO using EvoSpace. As a second validation alternative for our algorithm, we evaluated its performance against the Fuzzy Marine Predator Algorithm (FMPA) [27], a recent bioinspired algorithm with dynamic adaptation of parameters. For this comparison, we use the mathematical benchmark functions introduced in the Competition on Evolutionary Computation 2017 (CEC-2017) [58].

We also compare the LCGA with current state-of-the-art continuous optimization algorithms within the context of the CEC 2017 benchmarks by extending the comparison of Salgotra et al. [40], which included the algorithms SaDE [36], JADE [34], SHADE [35], MVMO [59], CV1.0, and CVnew.

The reader needs to note that while the black-box COCO Benchmark Framework and CEC-2017 benchmarks aim to represent the difficulties found in real-world optimization problems and are widely used in the evolutionary computation community, they may not fully capture the complexity and diversity of actual real-world optimization scenarios. Additionally, several “no free lunch” (NFL) theorems [60] establish that the performance of an optimization algorithm can be highly problem-specific. As a result, the transferability of algorithm performance from one problem or problem class to another is highly unpredictable and cannot be assumed.

### 4.1. Evaluation of Mathematical Benchmark Functions: COCO Framework

To obtain reliable results that allow us to evaluate the algorithm’s behavior and compare it against other algorithms, we consider it valuable and necessary to use an external tool: the COCO (COmparing Continuous Optimizers) Benchmark Framework. It is a platform for comparing continuous optimizers in a black box setup. Its goal is to automate the task of comparing numerical optimization algorithms. As an alternative for comparison, we will also use the evaluation results with the COCO reporting tool to visually compare the performance of our algorithm and other options.

We justify using the COCO Benchmark Framework based on the document *“The COCO Platform for Comparing Continuous Optimizers in a Black-Box Setting”* [57] by Hansen et al. This section outlines the chosen evaluation functions and dimensions for benchmarking. The evaluation extends to the COCO Benchmark Framework, encompassing 24 functions across dimensions 2 to 40. This external validation tool offers a broader perspective on LCGA’s performance and the results to compare with other established algorithms.

#### 4.1.1. Experimental Setup for COCO Benchmark Framework

This section details specifications for the COCO Benchmark Framework experiments, including the evaluation functions, dimensions, and instances. The random movement of optimal points adds a layer of robustness to the evaluation.

Below, in Table 1, we list the 24 evaluation functions that the COCO Benchmark Framework uses to obtain the comparison results of our algorithm, where it evaluated each function’s dimensions of 2, 3, 5, 10, 20, and 40. COCO evaluated 15 instances for each of these dimensions, and for each instance, the tool performed a random movement to relocate the optimal point of the function in the plane.

In the General Configuration shown in Table 2, we enumerate the configuration values used to run our research experiments for this tool. We chose these parameter values to balance exploration and exploitation, maintain diversity, and ensure robust and efficient optimization performance. We will describe each of the configuration parameters next.

- *Benchmark function*: Specifies that the algorithm will be tested across all available mathematical benchmark functions to ensure a comprehensive evaluation of its performance.
- *Population*: Indicates the population’s size at the algorithm’s first steps, meaning the number of individuals (potential solutions) generated at the population initialization step. A larger population can provide greater diversity but requires more computational resources.
- *Sample size*: Represents the number of individuals sampled from the population to apply certain genetic operations (or life-cycle steps), maintaining a manageable subset for operations while still reflecting the population’s diversity.
- *Evaluations*: Specifies the maximum number of fitness evaluations allowed, calculated as 10,000 times the problem’s dimensionality. This parameter controls the computational budget allocated for the optimization process.
- *Mutation rate*: Defines the rate or probability at which modifications are introduced in an individual each time it suffers the growth (or aging) step Section 3.3. A higher mutation rate can increase population’s genetic diversity and help escape local optima but disrupt convergence if it is too high.
- *Max age*: Sets the maximum age for individuals in the population. Once the individuals reach this age, they are removed by the death step, mimicking the natural life-cycle and ensuring continuous population turnover.
- *Survival Threshold*: Is another criteria used during the death step Section 3.5, to decide if an individual survives or dies. As shown in Equation (3), it is computed with the following elements: *Survive min*, *Survive max*, and *Pressure* in conjunction with the individual’s fitness. In combination, all three survival threshold elements control population pressure and avoid overcrowding.
  *Survive min*: This is the minimum survival rate, or the death’s initial relative fitness required by the survival threshold when compared to the best-found fitness.
  *Survive max*: This is the maximum survival rate, or the death’s final relative fitness required by the survival threshold when compared to the best-found fitness.
  *Pressure*: Refers to survival pressure and measures the intensity of death applied. Higher pressure favors the fittest individuals more strongly, potentially speeding up convergence but risking loss of diversity.

System Requirements Specification (SRS). To perform the experimental tests, we used a computer with the following hardware characteristics: processor Intel(R) Core(TM) i7-10750H CPU @ 2.60 GHz, minimum RAM required is 16 GB (we recommend 32 GB), and required space on the hard disk is 350 megabytes for COCO. The test computer’s software characteristics are Windows 11 Home Single Language, Version 23H2, System Type 64-bit OS, and x64-based processor. The programming language is Java, and the source code is in text files; therefore, any editor will be suitable to edit the source code. It requires installing (Java Development Kit (JDK) https://www.oracle.com/mx/java/technologies/downloads/, accessed on 10 July 2024) version 17.0.1 or above, with its corresponding configuration for the *system environment variables*. We used the default *Windows Command Prompt* application to compile the source code and run the tests. The instructions for compiling and running the code are available in the source-code README file at the (GitHub repository https://github.com/jcarlosfelix/lcga, accessed on 10 July 2024). Please consult the Data Availability Statement at the end of this document.

#### 4.1.2. COCO Benchmark Framework: LCGA Evaluation Results

**Figure 4:**
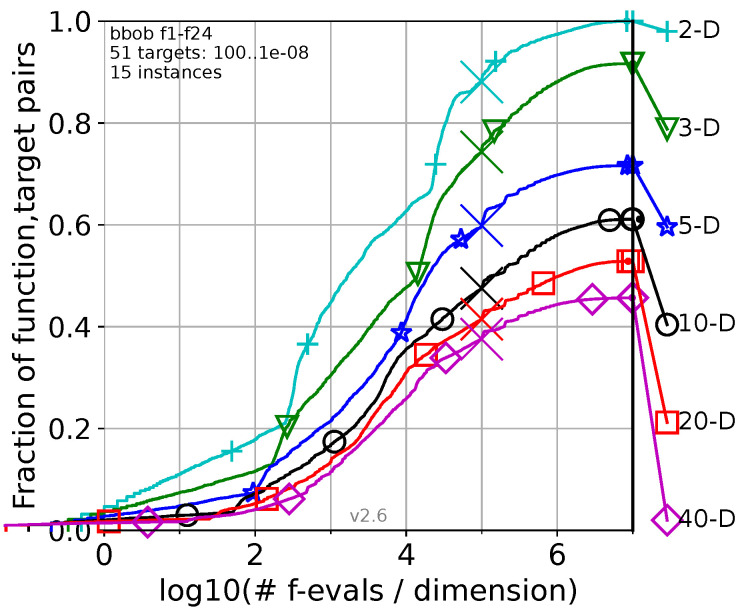
COCO Benchmark Framework: LCGA summary.

Graphical summaries generated by the COCO Benchmark Framework illustrate LCGA’s performance across 24 functions and multiple dimensions, where the evaluation results provide insights into the algorithm’s adaptability and efficiency. COCO generates Figure 4, where the graph summarizes the evaluation for the 24 functions of the COCO Benchmark Framework.

Figure 4 summarizes the evaluation results of the LCGA across 24 benchmark functions using the COCO framework. Next, we will explain how to interpret the results presented in the figure.

*X*-axis: log10(# f-evals/dimension). This axis represents the number of function evaluations per dimension, plotted on a logarithmic scale (base 10). It provides a normalized view of the computational effort required by the algorithm, making it easier to compare across different dimensions.

*Y*-axis: Fraction of function-target pairs that reached the target error level (1.00 × 10^−8^). This axis shows the fraction of function-target pairs for which the LCGA algorithm successfully reached the target error level (1.00 × 10^−8^). It indicates the algorithm’s success rate, with higher values representing better performance.

Each curve represents a different dimensionality (2D, 3D, 5D, 10D, 20D, and 40D). The curves show the LCGA’s performance overview across various dimensions, indicating how quickly and effectively it reaches the target error level as the number of evaluations increases. Higher curves indicate better performance, representing a more significant fraction of successful assessment for the given number of function evaluations. Steeper curves suggest rapid convergence to the target error level. The following is the figure’s plot annotations:

- f1–f24: Indicates that the performance data covers 24 benchmark functions from the COCO framework.
- 51 targets: 100..1 × 10^−8^: Refers to the range of target values (error levels) used in the evaluation, from 100 to 1 × 10^−8^.
- 15 instances: Denotes that each function evaluation was repeated 15 times to ensure robustness and reliability of the results.
- v2.6: Version of the COCO framework used for benchmarking.

#### 4.1.3. Comparative BBOB Plot

This section introduces the comparison plot showing the LCGA against GA [3], PSO [61], and EvoSpace [62], with subsequent sections for each comparison. This benchmark tool facilitates comparison analysis with previously evaluated algorithms; it presents the functionality of generating a graphical report utilizing evaluation data from the BBOB data archive. In the following section, we will show the comparison with the algorithms GA [63] (Genetic Algorithm 013), PSO [64] (Particle Swarm Optimization 026), and EvoSpace [65] (EvoSpace-PSO-GA 153), whose evaluation data results are available in the link https://numbbo.github.io/data-archive/bbob/, accessed on 10 July 2024.

The plot shows empirical runtime distributions, defined by the number of function evaluations divided by dimension. Because the number of evaluations increases with the dimension, the plot uses a logarithmic scale. The BBOB benchmark uses all functions with a range of target values (error) going from 100 to 1 × $10^{-8}$. The fraction of function-target pairs corresponds to the fraction of successful targets achieved within the maximum number of function evaluations, indicated by a cross. Algorithms with plots with a higher fraction and fewer function evaluations are better. Plots are shown for each dimension.

**Figure 5:**
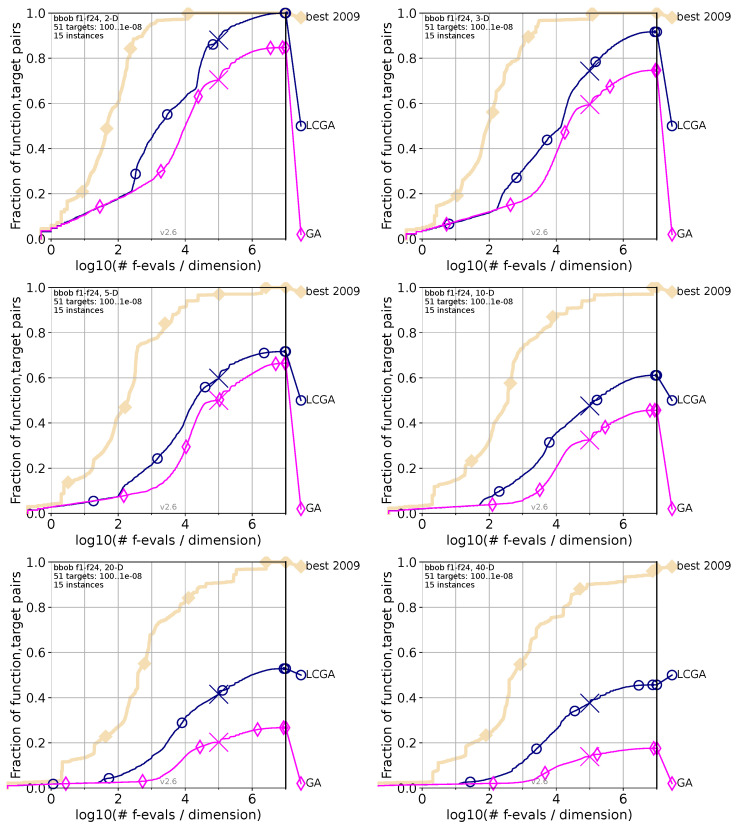
Comparative statistical analysis of the LCGA algorithm against the GA for dimensions 2, 3, 5, 10, 20, and 40, respectively, from top-left to right-bottom. On the *X*-axis, we will find the number of evaluations per dimension on a logarithmic scale of 10 for each graph. On the *Y*-axis, we will see the percentage of occasions in which the target error was found (target error 1.00 × $10^{-8}$). The curves show each algorithm’s performance overview, indicating how quickly and effectively it reaches the target error level as the number of evaluations increases. Higher curves indicate better performance, representing a more significant fraction of successful assessment for the given number of function evaluations. Steeper curves suggest rapid convergence to the target error level.

**Figure 6:**
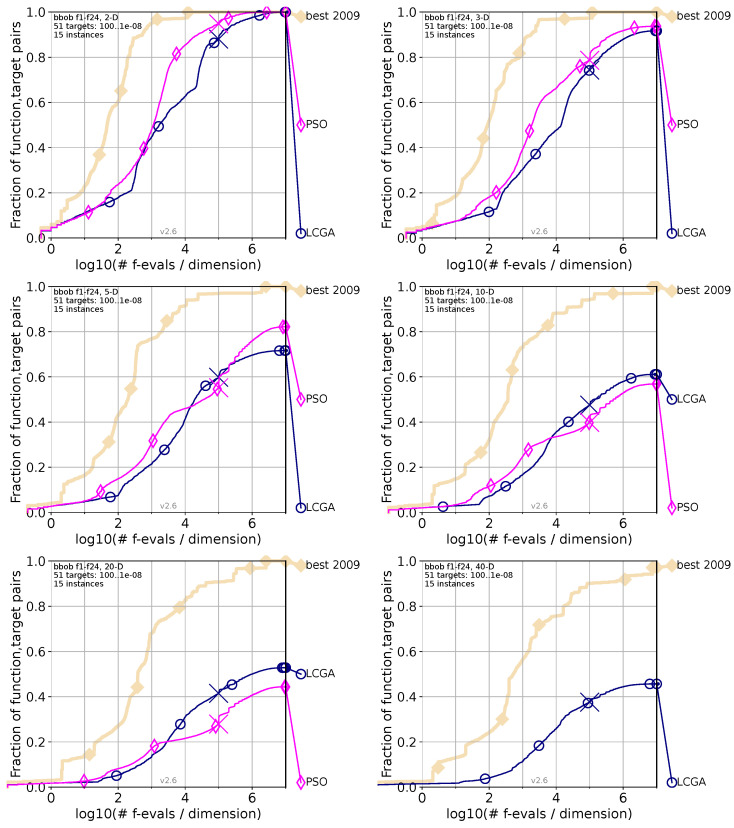
Comparative statistical analysis of the LCGA algorithm against the PSO for dimensions 2, 3, 5, 10, and 20, respectively, from top-left to right-bottom. On the *X*-axis, we will find the number of evaluations per dimension on a logarithmic scale of 10 for each graph. On the *Y*-axis, we will see the percentage of occasions in which the target error was found (target error 1.00 × $10^{-8}$). The curves show each algorithm’s performance overview, indicating how quickly and effectively it reaches the target error level as the number of evaluations increases. Higher curves indicate better performance, representing a more significant fraction of successful assessment for the given number of function evaluations. Steeper curves suggest rapid convergence to the target error level. As mentioned earlier, the figure does not include the results for the 40 dimensions of the PSO algorithm because the data were unavailable for said dimension.

**Figure 7:**
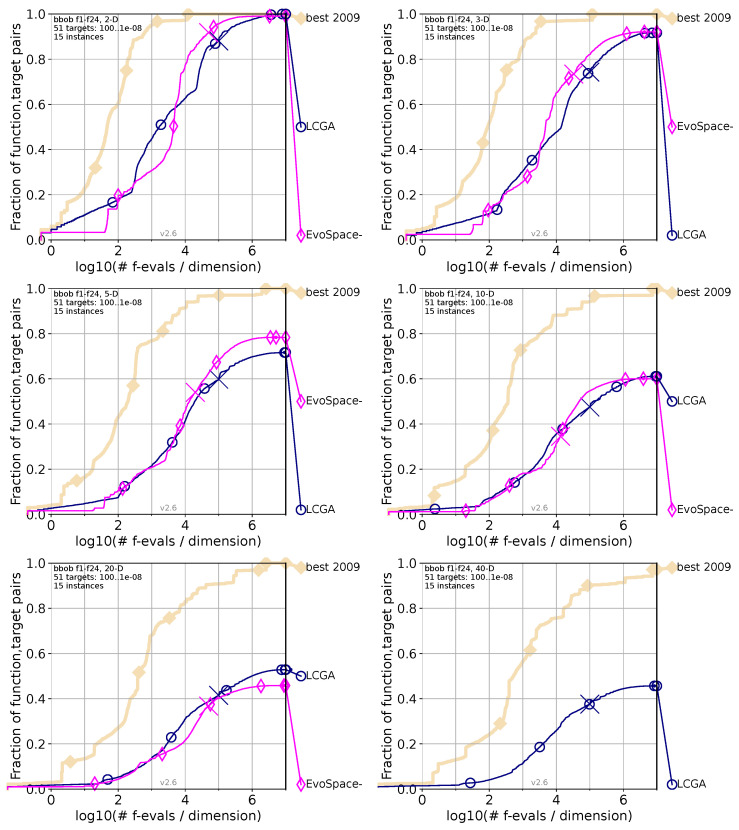
Comparative statistical analysis of the LCGA algorithm against the EvoSpace for dimensions 2, 3, 5, 10, and 20, respectively, from top-left to right-bottom. On the *X*-axis, we will find the number of evaluations per dimension on a logarithmic scale of 10 for each graph. On the *Y*-axis, we will see the percentage of occasions in which the target error was found (target error 1.00 × $10^{-8}$). The curves show each algorithm’s performance overview, indicating how quickly and effectively it reaches the target error level as the number of evaluations increases. Higher curves indicate better performance, representing a more significant fraction of successful assessment for the given number of function evaluations. Steeper curves suggest rapid convergence to the target error level. As mentioned earlier, the figure does not include the results for the 40 dimensions of the EvoSpace algorithm because the data were unavailable for said dimension.

Each of the following Figure 5, Figure 6 and Figure 7 compares the performance of the LCGA versus another specific algorithm across various dimensions using the COCO (Comparing Continuous Optimisers) benchmark framework. The navy blue marks our proposed LCGA, while the pink color is for the algorithm in comparison; as a reference, the beige marks the best algorithm evaluated in 2009 (best 2009). In the top-left corner are the two dimensions (2D); top-right corner for three dimensions (3D); middle-left for five dimensions (5D); middle-right for ten dimensions (10D); bottom-left corner 20 dimensions (20D); and bottom-right corner for 40 dimensions (40D).

It is critical to highlight that the comparative results for the **GA, PSO,** and **EvoSpace** algorithms experiment results are available at the following (COCO repository https://numbbo.github.io/data-archive/bbob/, accessed on 10 July 2024), with the identification **numbers 013, 026,** and **153**, respectively. One of the essential features of COCO is that before publishing, the COCO team verifies the legitimacy of the published results so they can have their validation and support. We must mention that in the following Figure 6 and Figure 7, we are missing the results for the 40 dimensions for the PSO and EvoSpace algorithms; we understand one of the reasons these results were unavailable is because the computers required too many computational resources to complete these tests, which at the time were unavailable to the researchers who published their work using this benchmark tool.

#### 4.1.4. LCGA Comparison with GA

Figure 5 compares the performance of the Life Cycle Genetic Algorithm (LCGA) and Genetic Algorithm (GA) across various dimensions using the COCO benchmark framework. The pink color marks GA, while navy blue is for LCGA. We can observe the following behaviors if we analyze each chart from the figure.

For the 2D chart, LCGA shows a rapid increase in the fraction of function-target pairs, achieving nearly 100 percent at around $10^{2}$ evaluations per dimension. At the same time, GA demonstrates a slower increase, not reaching the same level of success within the given number of examinations. In the 3D chart, LCGA and GA show a slower increase than in the 2D case. LCGA still outperforms GA, reaching higher success fractions faster. For the 5D chart, the performance gap between LCGA and GA widens, with LCGA achieving higher success rates quickly, while GA’s performance increase is much slower, indicating struggles with higher dimensions. In the 10D chart, LCGA continues to maintain a lead, although with a less steep increase compared to lower dimensions. GA displays a gradual performance improvement, but has yet to reach the success rates of LCGA. For the 20D chart, the success rate for both algorithms diminishes as dimensionality increases. LCGA’s success fraction peaks earlier than GA’s, suggesting better efficiency in higher dimensions. In the final 40D chart, LCGA and GA struggle in 40D space with significantly lower success fractions. Despite the lower success rate, LCGA outperforms GA, indicating a more robust performance in high-dimensional spaces.

Based on Figure 5, we can observe that the LCGA consistently outperforms the Genetic Algorithm (GA) across all dimensional spaces in the COCO benchmark framework. The LCGA’s ability to reach higher success fractions quickly, especially in lower dimensions, suggests it has a more efficient search and optimization process. As dimensions increase, both algorithms experience a decline in performance, but LCGA’s slower decline rate suggests it is better at managing the complexity of high-dimensional spaces. Despite the overall lower success rate, the robustness of LCGA in the 40D space indicates its potential utility in solving complex, high-dimensional problems where traditional algorithms like GA may falter. This performance advantage could be due to LCGA’s dynamic adaptation strategies, which include more effective mutation, crossover, and selection processes closer to the adaptive processes observed in natural evolution.

#### 4.1.5. LCGA Comparison with PSO

Figure 6 compares the performance of the Life Cycle Genetic Algorithm (LCGA) and Particle Swarm Optimization (PSO) across various dimensions using the COCO benchmark framework. The pink color marks PSO, while navy blue marks LCGA. We can observe the following behaviors if we analyze each chart from the figure.

For the 2D chart, PSO starts stronger than LCGA, maintaining its peak performance as the number of evaluations increases. PSO peaks earlier, indicating higher efficiency in 2D spaces. In the 3D chart, LCGA and PSO start similarly, but PSO again outperforms LCGA with fewer evaluations needed to reach a higher success rate. LCGA gradually increases, indicating it is less efficient than PSO in 3D spaces. For the 5D chart, the gap between LCGA and PSO reduces, with LCGA demonstrating a significantly higher success rate in a small chart segment. For the 10D chart, PSO initially leads, while LCGA gains momentum at the middle of the chart and flips, leading to never losing again. PSO’s performance lags LCGA, indicating potential scalability issues as dimensions grow. The 20D chart shows that the gap between LCGA and PSO widens in favor of LCGA. LCGA performs better, but as the problem space expands, both algorithms significantly reduce success rates as expected. PSO shows a more pronounced decline, reinforcing its scalability challenges. For the 40D chart, no data were available to compare for the PSO algorithm.

Based on Figure 6, the comparison between LCGA and PSO across various dimensions shows that PSO generally begins with a strong performance in lower dimensions (2D and 3D). Still, LCGA begins to outpace PSO as the complexity increases (5D and beyond). LCGA’s initial slower start suggests that exploring the solution space may take longer but eventually surpasses PSO’s early gains, particularly as the dimensions grow, suggesting better long-term efficiency and scalability. Without data for the 40D chart for PSO, we can infer that LCGA’s strategies could be more robust in high-dimensional spaces. This trend indicates that while PSO may be advantageous for quick optimization in more straightforward problems, LCGA’s approach might be more suitable for complex problems where a balance between exploration and exploitation is critical.

#### 4.1.6. LCGA Comparison with EvoSpace

Figure 7 compares the performance of the Life Cycle Genetic Algorithm (LCGA) and the EvoSpace algorithm across various dimensions using the COCO benchmark framework. The pink color marks EvoSpace, while navy blue marks LCGA. We can observe the following behaviors if we analyze each chart from the figure.

For the 2D chart, LCGA performs better at fewer evaluations. LCGA achieves a slightly higher success rate at about $10^{2}$ evaluations per dimension. In the 3D chart, EvoSpace surpasses LCGA in the latter half of the curve, indicating a higher efficiency. In the 5D chart, the gap between algorithms shows a slight dominance for EvoSpace at a higher number of evaluations for lower-dimension problems. For the 10D chart, the success rates for both algorithms begin to plateau, with LCGA leading, though by a smaller margin. EvoSpace shows a slower increase and, in the end, reaches a success rate similar to LCGA’s within the evaluation limits. In the 20D chart, LCGA maintains a higher success rate across the evaluations, indicating a steadier performance in higher dimensions. EvoSpace’s success rate is lower, showing that it may struggle more as the problem dimensionality increases. For the 40D chart, no data were available to compare for EvoSpace.

Based on Figure 7, the analysis of the COCO benchmark framework comparison between LCGA and EvoSpace across various dimensions suggests that EvoSpace may have an early advantage in lower dimensions but is overtaken by LCGA in specific scenarios, as it shows LCGA performs better as the problem complexity increases, particularly in 20D. The absence of data for EvoSpace in 40D prevents a direct comparison at this highest level of complexity. These trends imply that while EvoSpace might be more efficient in some mid-range dimensional spaces, LCGA potentially offers a more consistent and robust approach to higher-dimensional optimization challenges. The adaptability and scalability of LCGA could be more advantageous for complex problems where a strategic balance between exploration and exploitation over many evaluations is crucial.

#### 4.1.7. LCGA Comparison with Multiple GA Variants

**Figure 8:**
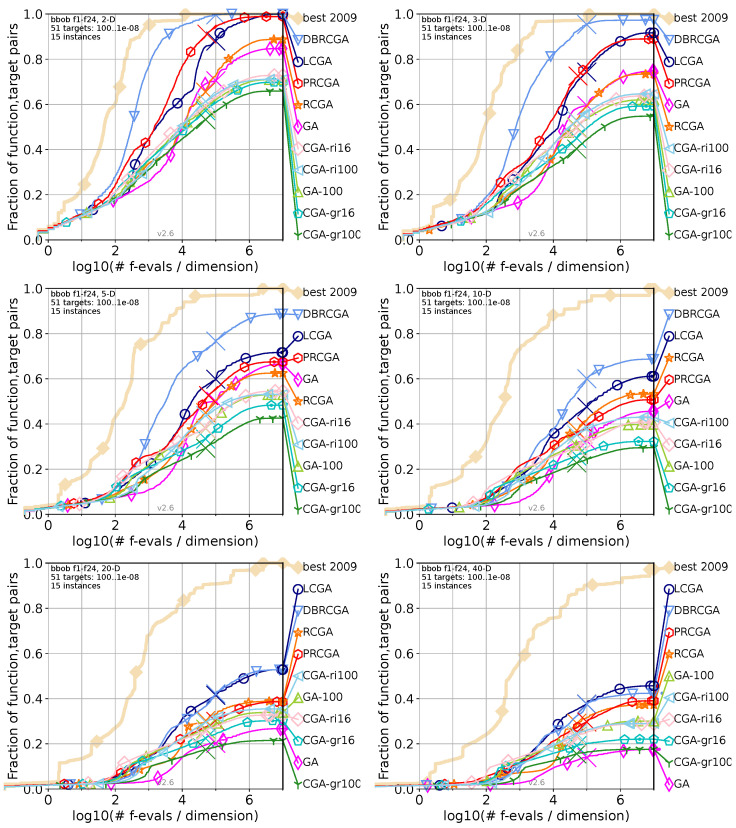
To demonstrate the effectiveness of the proposed strategy versus existing GA variants, we compare the statistical analysis of the LCGA algorithm against the GA and some of its improved derivate algorithms (available at the COCO repository https://numbbo.github.io/data-archive/bbob/, accessed on 10 July 2024). We display dimensions 2, 3, 5, 10, 20, and 40, respectively, from top-left to right-bottom. The curves show each algorithm’s performance overview, indicating how quickly and effectively it reaches the target error level as the number of evaluations increases. Higher curves indicate better performance, representing a more significant fraction of successful assessment for the given number of function evaluations. Steeper curves suggest rapid convergence to the target error level.

To validate and demonstrate the effectiveness of the proposed strategy versus existing GA variants, in the following Figure 8, we compare the statistical analysis of the LCGA algorithm against the GA and some of its improved derivate algorithms available at the (COCO repository https://numbbo.github.io/data-archive/bbob/, accessed on 10 July 2024). Next, we will briefly describe each reference algorithm.

- The Real-Coded Genetic Algorithm (RCGA) [66] employs floating-point representation for candidate solutions, enhancing its ability to solve complex real-valued optimization tasks. This algorithm utilizes tournament selection for parent selection, arithmetical crossover for recombination, and an adaptive-range variant of non-uniform mutation to introduce variability. The RCGA employs multiple independent restart mechanisms, which initiate a new optimization run whenever the algorithm meets its stopping criteria without carrying over any previous information.
- The Direction-Based Real-Coded Genetic Algorithm (DBRCGA) [67] enhances traditional real-coded genetic algorithms by incorporating a direction-based crossover (DBX) operator. This algorithm leverages relative fitness information to guide the crossover operation in a direction that significantly improves objective fitness. It employs a ranking selection (RS) mechanism to maintain population diversity and uses a dynamic random mutation (DRM) operator to introduce variability and prevent premature convergence.
- The Projection-Based Real-Coded Genetic Algorithm (PRCGA) [68] enhances the traditional RCGA by incorporating a projection-based exploratory search mechanism. This method projects a candidate solution onto a vector defined by the difference between two other solutions, effectively guiding the search toward promising regions of the solution space. PRCGA uses tournament selection for parent selection, blend-α crossover for recombination, and non-uniform mutation to introduce variability. Additionally, it includes a stagnation alleviation mechanism that refreshes a portion of the population when diversity drops below a threshold.
- The CGA-grid16 [69] algorithm is a cellular genetic algorithm in which the population is structured on a 4 × 4 grid (16 individuals). Each individual in the population has four neighbors in a north-east–west-south (NEWS) configuration. The algorithm uses a rank-based selection process for crossover and mutation, promoting diversity while facilitating the gradual spread of superior solutions throughout the grid.
- CGA-grid100 [69] is a variant of the cellular genetic algorithm with a larger population structured on a 10 × 10 grid (100 individuals). Like CGA-grid16, each individual has four neighbors (NEWS) and undergoes rank-based selection for crossover and mutation. The increased population size allows for greater exploration of the solution space.
- CGA-ring16 [69] employs a unidirectional ring topology for its population of 16 individuals. In this configuration, each individual has only one neighbor, forming a simple, linear ring. Due to the single-neighbor structure, the selection of mates is deterministic, simplifying the selection process and enhancing the spread of superior genes.
- CGA-ring100 [69] extends the ring topology to a larger population of 100 individuals. Each individual again has a single neighbor, forming a unidirectional ring. The deterministic selection process ensures efficient crossover and mutation operations. With the increased population size, CGA-ring100 benefits from enhanced exploration capabilities and maintains high diversity.
- The GA-100 [69] algorithm is a generational, single-population genetic algorithm with a population size of 100 individuals. It uses rank-based selection for choosing parents, followed by crossover and mutation to generate offspring. This algorithm aims to balance exploration and exploitation through its generational approach, where the entire population is replaced by offspring in each generation.

Analyzing LCGA’s performance across dimensions from Figure 8, the comparative analysis and the visual evidence lead us to the following statement. In the lower dimensions (2D and 3D), the Life Cycle Genetic Algorithm (LCGA) demonstrates rapid success. It consistently outperforms the traditional Genetic Algorithm (GA) and other variants, showcasing its efficiency in simpler problem spaces. As the dimensionality increases to 5D and 10D, LCGA maintains a lead over the different algorithms, with the gap narrowing slightly but still exhibiting faster convergence. In the 20D space, LCGA’s superior performance is evident as it achieves a higher success rate across the evaluations, indicating robust adaptability in more complex scenarios. Even in the highly challenging 40D space, where both LCGA and GA experience a decline in performance due to the increased complexity, LCGA still maintains a higher success rate, demonstrating its capability to handle complex, high-dimensional problems effectively.

As a comparative advantage, LCGA’s rapid and higher success in lower dimensions indicates a more efficient search and optimization process than traditional GA and its variants. As dimensionality increases, LCGA shows a slower decline in performance, suggesting superior management of high-dimensional spaces, likely due to its dynamic adaptation strategies in its life-cycle stages (or processes). This robustness and consistency across various dimensions highlight LCGA’s potential for complex optimization challenges. The algorithm’s ability to outperform other GA variants, particularly in higher-dimensional and more complicated problem spaces, underscores its effectiveness and reliability, making it a promising tool for solving intricate optimization tasks. In summary, the comparative analysis and the visual evidence from Figure 8 strongly support the superior performance of LCGA over traditional and enhanced GA variants, particularly in higher-dimensional spaces and complex optimization tasks.

### 4.2. Evaluation of CEC-2017 Reference Mathematical Functions

We perform comparison experiments using the reference mathematical functions presented in the CEC-2017 [58] to compare our proposal first against the Fuzzy Marine Predator Algorithm (FMPA) [27], where we can quantify the results so that through its statistical analysis, we can study the behavior of the algorithm in greater detail. Furthermore, we extend the comparison to other state-of-the-art continuous optimization algorithms. Experiments are conducted across 30 CEC-2017 mathematical functions, varying dimensions from 10 to 100. The LCGA algorithm, employing the continuous range crossover strategy, is compared with FMPA. Statistical analyses, including Z-tests, provide a robust comparative study. The choice of CEC-2017 mathematical functions is justified by referencing Awad et al. [58]. This section outlines the experimental setup, emphasizing the dimensions and the number of runs.

#### 4.2.1. Fuzzy Marine Predator Algorithm (FMPA)

The Marine Predator Algorithm (MPA) has been effectively utilized in various domains [39], showcasing improved outcomes over earlier approaches to diverse challenges [27]. These include forecasting COVID-19 spread and control systems design. Notable applications comprise integrating neuro-fuzzy models with MPA for enhanced biomethane production, employing hybrid intelligence methods for structural integrity assessment, optimizing photovoltaic system designs for maximal output under shading, refining fuzzy PID controller parameters, advancing digital image segmentation through multilevel thresholds and innovating X-ray image segmentation for swift COVID-19 detection in chest scans by leveraging a specialized MPA.

Researchers have integrated Fuzzy Logic Controllers (FLC) with traditional controls to boost system efficiency. Lately, there is a growing practice of optimizing fuzzy controllers using metaheuristics methods for ideal parametrization, reducing the target objective function. A recent work, the Fuzzy Marine Predator Algorithm (FMPA) [27], extends MPA with generalized type-2 fuzzy systems to adjust parameters. This algorithm has been benchmarked for its efficacy, notably in optimizing mobile robot controllers, showing improved solution quality and handling of uncertainties. It also serves as a comparative baseline for our proposal, using the FMPA as a comparative benchmark. We chose the FMPA as a reference because it is a recent bioinspired algorithm with dynamic adaptation of parameters that is used in real-world applications.

It is essential to mention that the complete test suite for CEC-2017 consists of 30 mathematical functions in the dimensions 10, 30, 50, and 100, for which we have provided the full results of our algorithm in Table 4 (LCGA Evaluation Results for the CEC-2017 Mathematical Functions), for the benefit of future publications, as other researchers will be able to compare their results against our algorithm proposal, contrast its findings, and highlight the improvements. In this particular case, when comparing our results with the FMPA algorithm publication [27], we only found published results for the first 19 functions. For this reason, the comparison against the algorithm above can only show the contrast for the 19 available benchmark evaluation functions, as shown in Tables 6–8 (comparative statistical analysis of the CEC-2017 mathematical functions evaluation).

#### 4.2.2. LCGA Experimental Setup

In our experiment section, we performed multiple tests to match and compare with the FMPA published results. In the following section, we outlined detailed experimental setups for LCGA, specifying dimensions, runs, and the Z-test statistical test with a 95 percent confidence level. The general configuration table shows the parameters used in the experiments. For each of the 30 CEC-2017 mathematical functions, we evaluated the 10, 30, 50, and 100 dimensions. We executed 51 runs for each dimension, according to the CEC specifications. We finish with the Z-test statistical test with a confidence level of 95 percent to determine the best alternative. We used the LCGA algorithm for our experiments, using the Continuous Range Crossover breeding strategy for reproduction mentioned in Section 3.4.

In the General Configuration shown in Table 3, we enumerate the configuration values used to run our research experiments for the CEC-2017 benchmark problems. We chose these parameter values to balance exploration and exploitation, maintain diversity, and ensure efficient optimization performance. Previously, we described each configuration parameter in Section 4.1.1.

System Requirements Specification (SRS). We used a computer with the hardware and software characteristics described earlier in Section 4.1.1 to perform the experimental tests, with the only difference being the hard space needed to store the results of all the CEC-2017 benchmark functions of nearly 200 gigabytes.

#### 4.2.3. LCGA Evaluation Results for the CEC-2017 Mathematical Functions

This section presents the evaluation results of LCGA across various mathematical functions and dimensions, showcasing its performance and providing a foundation for subsequent statistical analyses. The following Table 4, describes the LCGA evaluation results for the CEC-2017 mathematical functions for 10, 30, 50, and 100 dimensions.

#### 4.2.4. Statistical Analysis for the Comparative Study of the LCGA vs. FMPA Algorithms

Statistical analyses, including Z-tests, contribute to a comprehensive comparative study between LCGA and FMPA. Inferences drawn from these analyses explain the strengths and weaknesses of each algorithm under scrutiny. The emphasis is on the significance of statistical rigor in establishing the credibility of algorithmic comparisons. We used a left-tailed Z statistical test, found in Table 5. We display the Z-test equation in (4).

(4) $$Z=\frac{{\bar{x}}_{1}-{\bar{x}}_{2}}{\sqrt{\frac{\sigma_{1}^{2}}{n_{1}}+\frac{\sigma_{2}^{2}}{n_{2}}}}$$

Table 6 presents the comparative statistical analysis of the evaluation of CEC-2017 mathematical functions for 30 dimensions. Table 7 enlists the comparative results of the statistical study for the same benchmark for 50 dimensions. Finally, in Table 8, we can find the comparative statistical examination for the evaluation results for 100 dimensions.

#### 4.2.5. Comparative Results

To validate our research, we performed the statistical Z-test with a confidence level of 95 percent to analyze the alternatives we present in this proposal. To reduce uncertainty in the discovery of mathematical knowledge and to have greater precision in our comparison analysis, we consider studying the algorithms in both directions interesting. With the above, we mean the first of them is the one we showed in the previous Section 4.2.4, where for $\mu_{1}$, we use LCGA, and for $\mu_{2}$, we handle FMPA. In its counterpart, we do the inverse, where for $\mu_{1}$ we use FMPA, and for $\mu_{2}$, we use LCGA.

**Figure 9:**
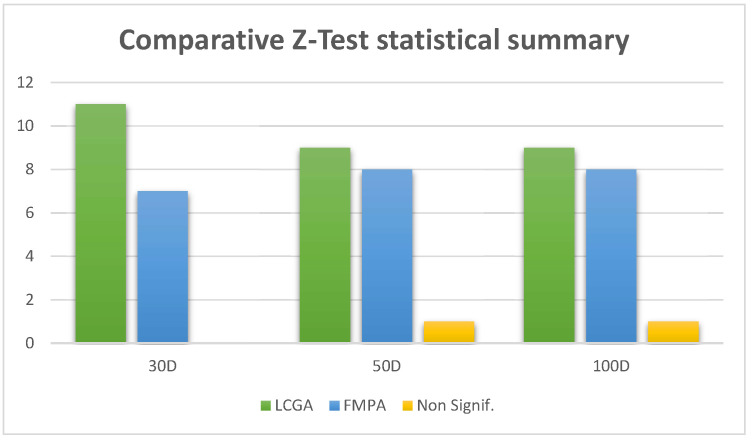
CEC-2017 statistical Z-test comparison: total number of times there was significant evidence that the algorithm was better with a 95 percent confidence.

Below, in Table 9, we show the comparative Z-test statistical summary for evaluating mathematical functions of the CEC-2017 for both LCGA and FMPA algorithms, with the total number of times there was significant evidence that the algorithm was better with a 95 percent confidence. The same table has a column named *Non-Signif.* that indicates the number of times the statistical analysis found no significant evidence to claim neither algorithm was better than its counterpart. The graphical representation of this data can be found in the following Figure 9, including 30, 50, and 100 dimensions.

The statistical comparison tables present the performance of LCGA against FMPA using the Z-test for the CEC-2017 benchmark in 30 dimensions. By analyzing the results shown, it is possible to make the following inferences: LCGA has a significant edge over FMPA in most of the functions tested; this is demonstrated by the win tallies (11 out of 18 for LCGA and 7 out of 18 for FMPA), indicating that LCGA is more effective in most benchmark functions. Moreover, the magnitude of the Z-scores shows considerable differences in mean scores between the two algorithms, with LCGA frequently achieving better optimization results with lower standard deviations, hinting at its consistent performance. These results suggest that LCGA might be a preferable algorithm for problems represented within the CEC-2017 benchmark, particularly when seeking reliable performance across multiple runs and diverse optimization problems.

The table comparing LCGA and FMPA for the CEC-2017 benchmark in 50 dimensions shows a nearly even split in wins between the algorithms, with LCGA winning 9 of 18 instances and FMPA winning 8 of 18 benchmark functions; this suggests that both algorithms have comparable performance across a range of complex, high-dimensional optimization problems. However, the individual Z-scores and mean values indicate that the differences in performance can be substantial depending on the specific function chosen for optimization. The standard deviations suggest that the reliability of each algorithm may vary, with some functions showing more variability in the results. Overall, the choice between LCGA and FMPA may depend on the specific characteristics of the problem at hand, with neither algorithm showing a definitive edge across all tested functions.

The comparison between LCGA and FMPA for the CEC-2017 benchmark in 100 dimensions shows LCGA slightly outperforming FMPA in 9 out of 18 functions. LCGA’s wins are distributed across a range of functions, suggesting versatility. In contrast, FMPA has a stronger showing in 8 out of 18 functions. Notably, the large Z-scores in some functions indicate significant performance differences. The high mean and standard deviation values in certain functions for both algorithms suggest variable performance, which might reflect the complexity inherent in the 100-dimensional search space. This evaluation highlights the importance of selecting an appropriate optimization algorithm based on the specific nature and dimensionality of the problem. Through statistical analysis, our proposed strategy is considered an excellent alternative to the marine algorithm improved with a type 2 generalized fuzzy system.

### 4.3. State-of-the-Art: Evaluation of CEC-2017 Mathematical Benchmark Functions

In their research, Salgotra et al. [40] presented a detailed analysis and enhancement of the Cuckoo Search (CS) algorithm, introducing an improved version named *CSsin*. This enhanced version addresses several key areas to optimize its performance on the CEC 2017 and CEC 2020 benchmark problems, known for their complexity and diversity. We will briefly enumerate some critical enhancements in CSsin in the following paragraph. For the *global search technique*, the CSsin algorithm employs a new Cauchy-distributed global search during the initial iterations, which aids in extensive search space exploration, preventing the algorithm from getting trapped in local optima. The global search equation leverages the Cauchy distribution’s fat-tailed property to make larger steps, enhancing the search process’s global exploration capabilities. For the *local search technique*, CSsin uses a sinusoidal adaptive decreasing adjustment inspired by the LSHADE-cnEpSin algorithm. This adjustment ensures efficient local exploitation, refining solutions within promising regions of the search space.

Another essential feature is CSsin incorporates a dual search strategy to balance exploration (searching new areas) and exploitation (refining known good areas). This strategy helps achieve a dynamic balance between the two phases, improving overall optimization performance. On the other hand, another of its features is that it uses a linearly decreasing switch probability to transition smoothly from exploration to exploitation over iterations. This gradual decrease ensures that the algorithm initially explores broadly and then focuses on exploiting the best solutions found. This strategy promotes linearly decreasing the population size with iterations to reduce the computational burden. This method maintains diversity in the early stages and enhances convergence speed in the later stages by focusing computational resources on the most promising solutions.

**State-of-the-Art Comparison**.

- SaDE: the Self-adaptive Differential Evolution with Neighborhood Search (SaDE) [36];
- JADE: the Adaptive Differential Evolution with Optional External Archive [34];
- SHADE: the Success-History-Based Parameter Adaptation for Differential Evolution (SHADE) [35];
- MVMO: The Determination of Dynamic Wind Farm Equivalents using Heuristic Optimization [59];
- CV1.0 and CVnew: CV1.0 uses Cauchy and Normal distributions to enhance exploration and exploitation, while CVnew introduces parameter adaptation and population reduction strategies [40];
- RB-IPOP-CMA-ES: Another state-of-the-art algorithm to consider is the IPOP-CMA-ES and its improved version RB-IPOP-CMA-ES [31].

**Statistical Significance**. To validate our research, we performed the statistical Z-test with a 95 percent confidence level to analyze the alternatives we present in this proposal. In the following Table 10, Table 11 and Table 12, we display the comparative Z-test statistical summary for evaluating 30 mathematical functions of the CEC-2017 benchmark problems to indicate if LCGA is significantly better than or competitive with state-of-the-art algorithms. Each column shows the statistical values for an algorithm, where we group each of the 30 benchmark functions in three rows: mean (Mean), Standard Deviation (St.Dv), and comparison result (Comp.).

According to our Z-test with a 95 percent confidence level, there are three possible comparison values: “-” means that our proposed LCGA performs better than the algorithm under consideration; “+” represents the opposite; and “=” indicates that the statistical analysis found no significant evidence to claim neither algorithm was better than its counterpart. On the last row for each column, the comparison value is displayed in terms of win/tie/loss (w/t/l), where win gives the accumulated minus (“-”), highlighted in green, the tie gives the total number of equals (“=”) found, and loss shows the accumulated plus (“+”) counts highlighted in red.

In Table 10, Table 11 and Table 12, we display the CEC-2017 statistical results for 50 dimensions for the benchmark functions 1 to 10, 11 to 20, and 21 to 30, respectively. Table 13 displays the CEC-2017 summarized statistical results for all 50D functions (1 to 30).

## 5. Discussion

In our statistical comparison, we draw the following conclusions based on the COCO benchmark framework results analysis comparing LCGA with the GA, PSO, and EvoSpace algorithms. The COCO experiment analysis indicates that the LCGA demonstrates superior performance relative to the GA within the entire range of dimensional spaces tested in the COCO benchmark framework. LCGA exhibits a marked proficiency in attaining elevated success ratios swifter, particularly in reduced dimensional scenarios, indicative of its superior search efficacy and optimization capabilities in this context. Although a performance differential is evident for both algorithms in increased dimensionality, LCGA manifests a more gradual performance deterioration. This resilience hints at its enhanced ability to navigate the complexities of optimizing in extensive multi-dimensional landscapes.

In the COCO comparative study of LCGA and PSO, the analysis across a spectrum of dimensions reveals that while PSO typically exhibits robust early performance in simpler two- and three-dimensional spaces, LCGA gains a competitive edge in more complex scenarios, starting from five dimensions onward. The results’ analysis indicates that while LCGA may take more time initially to sample the solution space, its performance trajectory exceeds that of PSO’s quick outset, especially as the problem’s complexity escalates. Such observations imply that LCGA may possess superior long-term efficiency and scalability when tackling increasing dimensionality problems.

The comparative study between LCGA and EvoSpace for the COCO benchmark framework reveals an initial dominance by EvoSpace in lower-dimensional settings, which subsequently cedes ground to LCGA in certain conditions: LCGA’s enhanced performance with increased problem complexities, notably in the 20D context. This observation suggests that while EvoSpace may excel within mid-dimensional ranges, LCGA may deliver a more uniform and resilient strategy when confronted with high-dimensional optimization challenges. LCGA’s adaptability and scalability could present significant advantages in complex scenarios that demand a nuanced equilibrium between exploration and exploitation throughout extensive evaluation processes.

Moving on to the second experiment, using the CEC-2017 benchmark across multiple dimensions for our statistical comparison, the LCGA performance against the Fuzzy Marine Predator Algorithm (FMPA) reveals distinct outcomes based on problem complexity and dimensionality. At the 30-dimensional level, LCGA shows a clear advantage, winning 11 out of 18 benchmark functions. Its efficacy in handling various optimization challenges with greater consistency and reduced variability in results demonstrates that LCGA can be relied upon for robust performance across diverse scenarios. Moving to 50 dimensions, the competition between LCGA and FMPA becomes more evenly matched, with each algorithm excelling in roughly half of the test cases. Suggesting that while LCGA maintains strong performance, FMPA catches up in certain functions. Choosing between the two will depend on specific problem characteristics and optimization needs. The near-even split highlights that both algorithms can adapt to complex, high-dimensional spaces, but their effectiveness can vary significantly depending on the challenge function for optimization.

In the 100-dimensional tests, LCGA displays its versatility by outperforming FMPA in most functions, underscoring its capacity to adapt to high-dimensional and complex problem spaces. Despite this, both algorithms exhibit high variability in their results, attributed to the inherent challenges of navigating expansive search spaces. This variability emphasizes the importance of selecting an optimization algorithm that addresses the problem’s specific nature and can manage the increased complexity introduced by higher dimensions. Overall, the comprehensive statistical analysis underscores LCGA’s potential as a superior alternative to the marine algorithm improved with a type-2 generalized fuzzy system, particularly in scenarios requiring dependable performance across various complex and varied optimization problems. The data support LCGA’s suitability for complex environments where the strategic balance between exploration and exploitation is crucial for achieving optimal solutions.

## 6. Conclusions and Future Work

This paper aims to provide a comprehensive overview of the Life Cycle Genetic Algorithm, its design rationale, experimental evaluations, and comparative analyses against existing algorithms. The LCGA algorithm mimics the life cycle stages of animal—birth, growth, reproduction, and death—to find the optimum of complex real-valued functions, including multimodal ones. Moreover, the document details the algorithm’s structure, stages, and comparative performance against established benchmarks, highlighting its potential for addressing complex optimization problems.

As future work for this line of research, we can mention the following alternatives: implementing the algorithm in the cloud to confirm whether the performance or precision of the results obtained by this different implementation strategy improves. Likewise, we could explore the algorithm’s behavior when working on the same computer in a multi-thread implementation technique, taking advantage of the architecture of several cores in a processor.

## Figures and Tables

**Table 1:** COCO evaluation functions.

Fx	Function Name
f1	Sphere
f2	Ellipsoid separable
f3	Rastrigin separable
f4	Skew Rastrigin-Bueche separable
f5	Linear slope
f6	Attractive sector
f7	Step-ellipsoid
f8	Rosenbrock original
f9	Rosenbrock rotated
f10	Ellipsoid
f11	Discus
f12	Bent cigar
f13	Sharp ridge
f14	Sum of different powers
f15	Rastrigin
f16	Weierstrass
f17	Schaffer F7, condition 10
f18	Schaffer F7, condition 1000
f19	Griewank-Rosenbrock F8F2
f20	Schwefel x * sin(x)
f21	Gallagher 101 peaks
f22	Gallagher 21 peaks
f23	Katsuura
f24	Lunacek bi-Rastrigin

**Table 2:** COCO—LCGA general configuration.

Configuration	Value
Benchmark function	ALL
Population	500
Sample size	20
Evaluations	10,000 * Dim
Mutation rate	7
Max age	40
Survive min	80
Survive max	100
Pressure	6

**Table 3:** LCGA general configuration.

Configuration	Value
Benchmark function	ALL
Population	500
Sample size	20
Evaluations	10,000 * Dim
Mutation rate	7
Max age	5
Survive min	80
Survive max	100
Pressure	6

**Table 4:** LCGA evaluation results for the CEC-2017 mathematical functions.

LCGA	CEC-2017 . 10D		CEC-2017 . 30D		CEC-2017 . 50D		CEC-2017 . 100D	
Fx	Mean	Std. Dev.	Mean	Std. Dev.	Mean	Std. Dev.	Mean	Std. Dev.
F1	1.1944E+03	1.4963E+03	4.1209E+03	3.7733E+03	2.4089E+03	3.0316E+03	6.2414E+03	4.8433E+03
F2	3.1589E+02	1.2929E+03	4.8743E+10	9.6983E+10	1.1921E+15	7.9339E+15	2.4258E+40	1.5841E+41
F3	3.4086E+02	7.0737E+02	3.1160E+03	2.1122E+03	1.0477E+04	4.0503E+03	4.6086E+04	9.4932E+03
F4	7.5915E+00	1.3342E+01	9.2684E+01	2.0569E+01	1.2352E+02	5.7181E+01	3.1222E+02	5.8262E+01
F5	9.4707E+00	3.5336E+00	8.0514E+01	1.8340E+01	1.8301E+02	2.5057E+01	5.5042E+02	4.9878E+01
F6	1.3253E-02	2.6043E-02	3.1026E-02	4.8007E-02	3.9868E-02	4.5024E-02	3.8646E-02	2.3243E-02
F7	2.4902E+01	6.8532E+00	1.5288E+02	3.0759E+01	3.2224E+02	5.6997E+01	9.2211E+02	1.1134E+02
F8	7.8721E+00	3.1877E+00	7.3613E+01	1.6509E+01	1.7896E+02	3.0522E+01	5.7996E+02	6.0357E+01
F9	9.9384E+00	1.3370E+01	7.6487E+02	4.1142E+02	3.2164E+03	1.0212E+03	1.3728E+04	2.0331E+03
F10	5.1135E+02	2.4348E+02	2.8804E+03	5.4171E+02	4.8975E+03	7.5793E+02	1.1883E+04	1.1807E+03
F11	1.1628E+01	5.8848E+00	9.7325E+01	3.6948E+01	3.1707E+02	3.4108E+02	2.1841E+03	1.1782E+03
F12	1.0486E+05	3.3360E+05	1.2479E+06	9.1424E+05	3.3163E+06	1.7724E+06	1.3494E+07	5.8015E+06
F13	6.9911E+03	6.3627E+03	9.2334E+03	7.8685E+03	2.2810E+03	2.5780E+03	2.9032E+03	1.9734E+03
F14	1.8604E+03	3.1363E+03	2.7176E+05	2.8365E+05	1.0176E+06	9.6677E+05	1.6446E+06	7.3152E+05
F15	2.8548E+03	4.1876E+03	3.5396E+03	4.7118E+03	3.4140E+03	3.5109E+03	2.0752E+03	2.2205E+03
F16	1.4380E+02	9.0688E+01	1.2422E+03	2.7231E+02	1.8152E+03	4.8596E+02	4.4579E+03	5.5859E+02
F17	1.3496E+01	1.3512E+01	6.1596E+02	1.7523E+02	1.4731E+03	3.8094E+02	3.3357E+03	4.9967E+02
F18	7.1687E+03	6.0118E+03	9.2606E+05	1.2131E+06	2.3988E+06	1.2071E+06	1.5574E+06	7.8375E+05
F19	3.5845E+03	4.4515E+03	5.0993E+03	5.7244E+03	1.1802E+04	6.3737E+03	1.1868E+03	1.2703E+03
F20	5.4050E+00	5.3584E+00	4.4557E+02	2.0099E+02	1.1021E+03	3.0317E+02	3.0783E+03	5.8148E+02
F21	1.4766E+02	5.2493E+01	2.7832E+02	1.7123E+01	3.9174E+02	3.7305E+01	7.9039E+02	5.9096E+01
F22	1.0715E+02	4.7288E+00	1.5949E+02	4.1872E+02	5.4797E+03	1.5516E+03	1.3071E+04	2.2196E+03
F23	3.2240E+02	7.8443E+00	4.9030E+02	3.9744E+01	7.6391E+02	8.7777E+01	9.8955E+02	7.1272E+01
F24	3.0737E+02	9.7077E+01	6.8270E+02	7.1288E+01	1.0985E+03	1.2919E+02	1.7787E+03	1.3972E+02
F25	4.3426E+02	2.2304E+01	4.0106E+02	2.1026E+01	5.6636E+02	2.3436E+01	8.2694E+02	5.1965E+01
F26	3.6661E+02	9.7987E+01	2.3078E+03	1.0223E+03	4.8879E+03	9.5080E+02	1.3429E+04	3.3451E+03
F27	3.9134E+02	9.0523E+00	4.9104E+02	1.7150E+01	5.8851E+02	1.4963E+02	7.8407E+02	1.7421E+02
F28	4.0440E+02	9.5670E+01	4.3487E+02	2.5479E+01	5.4744E+02	3.7986E+01	6.4166E+02	5.9264E+01
F29	2.9154E+02	2.6736E+01	8.0196E+02	1.7880E+02	1.2022E+03	3.0951E+02	3.3027E+03	4.0229E+02
F30	3.3099E+04	6.8265E+04	1.1008E+03	1.3508E+03	3.2905E+03	3.3981E+03	5.2712E+03	3.8947E+03

**Table 5:** Left-tailed Z statistical test.

Parameter Name	Value
Significance Level	0.05
Rejection Region	[−*∞* : −1.6449]
Acceptance Region	[−1.6449 : *∞*]
n	51
Null hypothesis	Ho: $\mu_{1}$ > $\mu_{2}$
Alternative hypothesis	Ha: $\mu_{1}$ < $\mu_{2}$
$\mu_{1}$	LCGA
$\mu_{2}$	VERSUS

**Table 6:** Comparative statistical analysis of the CEC-2017 mathematical functions evaluation, 30 dimensions.

CEC-2017 . 30D		LCGA		FMPA	
Fx	Z	Mean	Std. Dev.	Mean	Std. Dev.
F1	**−5.39E+00**	4.12E+03	3.77E+03	2.53E+07	2.57E+07
F2	*NA*	4.87E+10	9.70E+10	*NA*	*NA*
F3	9.52E+00	3.12E+03	2.11E+03	3.00E+02	0.00E+00
F4	**−1.07E+02**	9.27E+01	2.06E+01	4.00E+02	0.00E+00
F5	**−1.63E+02**	8.05E+01	1.83E+01	5.00E+02	0.00E+00
F6	**−7.81E+02**	3.10E−02	4.80E−02	6.06E+02	4.25E+00
F7	**−8.34E+01**	1.53E+02	3.08E+01	7.39E+02	3.04E+01
F8	**−3.14E+02**	7.36E+01	1.65E+01	8.00E+02	0.00E+00
F9	**−2.08E+03**	7.65E+02	4.11E+02	1.23E+05	6.51E+01
F10	2.48E+01	2.88E+03	5.42E+02	1.00E+03	0.00E+00
F11	**−1.16E+02**	9.73E+01	3.69E+01	1.23E+03	4.56E+01
F12	**−3.54E+00**	1.25E+06	9.14E+05	2.13E+06	1.17E+06
F13	7.00E+00	9.23E+03	7.87E+03	1.51E+03	2.87E+02
F14	6.80E+00	2.72E+05	2.84E+05	1.61E+03	2.38E+01
F15	3.00E+00	3.54E+03	4.71E+03	1.56E+03	3.18E+01
F16	**−1.05E+01**	1.24E+03	2.72E+02	1.70E+03	1.15E+02
F17	**−4.42E+01**	6.16E+02	1.75E+02	1.87E+03	7.79E+01
F18	5.44E+00	9.26E+05	1.21E+06	1.86E+03	4.11E+01
F19	3.93E+00	5.10E+03	5.72E+03	1.95E+03	4.37E+01
**Total (W)**	**11 of 18**	**$\mu_{1}$: LCGA**		**$\mu_{2}$: GT2FLS**	

**Table 7:** Comparative statistical analysis of the CEC-2017 mathematical functions evaluation, 50 dimensions.

CEC-2017 . 50D		LCGA		FMPA	
Fx	Z	Mean	Std. Dev.	Mean	Std. Dev.
F1	5.39E+00	2.41E+03	3.03E+03	1.19E+02	8.02E+01
F2	*NA*	1.19E+15	7.93E+15	*NA*	*NA*
F3	1.79E+01	1.05E+04	4.05E+03	3.00E+02	0.00E+00
F4	**−3.45E+01**	1.24E+02	5.72E+01	4.00E+02	0.00E+00
F5	**−9.03E+01**	1.83E+02	2.51E+01	5.00E+02	0.00E+00
F6	**−5.29E+02**	3.99E−02	4.50E−02	6.11E+02	6.32E+00
F7	**−7.16E+01**	3.22E+02	5.70E+01	9.19E+02	1.31E+01
F8	**−1.45E+02**	1.79E+02	3.05E+01	8.00E+02	0.00E+00
F9	**−1.91E+00**	3.22E+03	1.02E+03	3.51E+03	3.07E+02
F10	3.67E+01	4.90E+03	7.58E+02	1.00E+03	0.00E+00
F11	**−2.11E+01**	3.17E+02	3.41E+02	1.33E+03	2.17E+01
F12	1.34E+01	3.32E+06	1.77E+06	1.68E+03	2.73E+02
F13	3.03E−02	2.28E+03	2.58E+03	2.26E+03	3.24E+03
F14	7.51E+00	1.02E+06	9.67E+05	1.51E+03	6.82E+01
F15	3.73E+00	3.41E+03	3.51E+03	1.58E+03	7.15E+01
F16	**−2.30E+00**	1.82E+03	4.86E+02	1.99E+03	1.84E+02
F17	**−5.45E+00**	1.47E+03	3.81E+02	1.77E+03	6.00E+01
F18	1.42E+01	2.40E+06	1.21E+06	1.83E+03	3.06E+01
F19	7.17E+00	1.18E+04	6.37E+03	3.10E+03	4.51E+03
**Total (W)**	**9 of 18**	**$\mu_{1}$: LCGA**		**$\mu_{2}$: GT2FLS**	

**Table 8:** Comparative statistical analysis of the CEC-2017 mathematical functions evaluation, 100 dimensions.

CEC-2017 . 100D		LCGA		FMPA	
**Fx**	**Z**	**Mean**	**Std. Dev.**	**Mean**	**Std. Dev.**
F1	**−8.74E+01**	6.24E+03	4.84E+03	6.55E+04	0.00E+00
F2	*NA*	2.43E+40	1.58E+41	*NA*	*NA*
F3	3.44E+01	4.61E+04	9.49E+03	3.00E+02	0.00E+00
F4	**−1.08E+01**	3.12E+02	5.83E+01	4.00E+02	0.00E+00
F5	7.22E+00	5.50E+02	4.99E+01	5.00E+02	0.00E+00
F6	**−5.27E+02**	3.86E−02	2.32E−02	6.75E+02	7.02E+00
F7	4.44E−01	9.22E+02	1.11E+02	9.02E+02	2.33E+02
F8	**−2.60E+01**	5.80E+02	6.04E+01	8.00E+02	0.00E+00
F9	4.50E+01	1.37E+04	2.03E+03	9.10E+02	1.18E−13
F10	6.58E+01	1.19E+04	1.18E+03	1.00E+03	0.00E+00
F11	4.80E+00	2.18E+03	1.18E+03	1.39E+03	5.43E+01
F12	**−1.25E+01**	1.35E+07	5.80E+06	1.10E+09	4.75E+08
F13	**−9.18E+00**	2.90E+03	1.97E+03	1.86E+07	1.11E+07
F14	1.60E+01	1.64E+06	7.32E+05	6.70E+03	2.59E+03
F15	**−8.74E+00**	2.08E+03	2.22E+03	1.40E+06	8.76E+05
F16	2.58E+00	4.46E+03	5.59E+02	2.65E+03	3.82E+03
F17	**−1.65E+01**	3.34E+03	5.00E+02	5.19E+03	4.81E+02
F18	6.45E+00	1.56E+06	7.84E+05	7.14E+05	3.89E+05
F19	**−1.34E+01**	1.19E+03	1.27E+03	5.28E+06	2.15E+06
**Total (W)**	**9 of 18**	**$\mu_{1}$: LCGA**		**$\mu_{2}$: GT2FLS**	

**Table 9:** Comparative Z-test statistical summary for the evaluation of mathematical functions of the CEC-2017.

Dim	Z-test	LCGA	FMPA	Non-Signif.
30D	Total (W)	11/18	7/18	0/18
50D	Total (W)	9/18	8/18	1/18
100D	Total (W)	9/18	8/18	1/18

**Table 10:** CEC-2017 statistical results for 50D, benchmark functions: 1 to 10. There are three possible comparison values: “-” means that our proposed LCGA performs better than the algorithm under consideration; “+” represents the opposite; and “=” indicates that the statistical analysis found no significant evidence to claim neither algorithm was better than its counterpart. On the last row for each column, the comparison value is displayed in terms of win/tie/loss (w/t/l), where win gives the accumulated minus (“-”) highlighted in green, the tie gives the total number of equals (“=”) found, and loss shows the accumulated plus (“+”) counts highlighted in red.

Fx	Stats	SaDE	JADE	SHADE	MVMO	CV1.0	CVnew	CSsin	RB-IPOP-CMA-ES	LCGA
**F1**	**Mean**	1.21E+03	5.23E-14	0.00E+00	1.33E-05	1.00E+10	1.00E+10	1.00E+10	1.13E-07	2.41E+03
	**St.Dv**	1.97E+03	2.51E-14	0.00E+00	5.60E-06	0.00E+00	0.00E+00	0.00E+00	4.26E-08	3.03E+03
	**Comp.**	+	+	+	+	-	-	-	+	
**F2**	**Mean**	9.27E+01	1.31E+13	1.08E+12	1.80E+17	1.00E+10	1.00E+10	1.00E+10	2.77E+05	1.19E+15
	**St.Dv**	4.12E+01	8.53E+13	4.39E+12	1.27E+18	0.00E+00	0.00E+00	0.00E+00	1.98E+06	7.93E+15
	**Comp.**	=	=	=	=	=	=	=	=	
**F3**	**Mean**	2.71E+02	1.77E+04	0.00E+00	5.30E-07	1.95E+04	8.71E+04	1.07E+04	0.00E+00	1.05E+04
	**St.Dv**	8.28E+02	3.70E+04	0.00E+00	1.09E-07	6.27E+03	4.08E+03	6.68E+03	0.00E+00	4.05E+03
	**Comp.**	+	=	+	+	-	-	=	+	
**F4**	**Mean**	8.92E+01	4.96E+01	5.68E+01	3.58E+01	1.16E+02	2.67E+01	1.88E+01	2.96E+01	1.24E+02
	**St.Dv**	4.21E+01	4.71E+01	8.80E+00	3.66E+01	6.27E+03	5.92E+00	3.45E+01	4.07E+01	5.72E+01
	**Comp.**	+	+	+	+	=	+	+	+	
**F5**	**Mean**	9.23E+01	5.42E+01	3.28E+01	8.07E+01	3.41E+02	2.39E+02	3.09E+02	2.79E+00	1.83E+02
	**St.Dv**	1.86E+01	8.80E+00	5.03E+00	1.64E+01	8.02E+01	3.80E+01	2.10E+01	1.44E+00	2.51E+01
	**Comp.**	+	+	+	+	-	-	-	+	
**F6**	**Mean**	7.43E-03	1.44E-13	8.38E-04	5.43E-03	4.85E+01	4.07E+01	1.00E+01	1.63E-07	3.99E-02
	**St.Dv**	2.35E-02	9.11E-14	1.01E-03	3.30E-03	4.85E+01	8.14E+00	5.20E+00	1.38E-07	4.50E-02
	**Comp.**	+	+	+	+	-	-	-	+	
**F7**	**Mean**	1.40E+02	1.01E+02	8.09E+01	1.23E+02	2.74E+02	2.22E+02	1.39E+02	5.66E+01	3.22E+02
	**St.Dv**	1.97E+01	6.48E+00	3.78E+00	1.27E+01	7.29E+01	3.49E+01	9.71E+01	1.39E+00	5.70E+01
	**Comp.**	+	+	+	+	+	+	+	+	
**F8**	**Mean**	9.42E+01	5.52E+01	3.23E+01	7.59E+01	3.29E+02	2.50E+02	3.17E+02	2.58E+00	1.79E+02
	**St.Dv**	1.77E+01	7.76E+00	3.82E+00	1.61E+01	7.29E+01	4.51E+01	2.43E+01	1.79E+00	3.05E+01
	**Comp.**	+	+	+	+	-	-	-	+	
**F9**	**Mean**	4.83E+01	1.17E+00	1.11E+00	7.38E+00	1.00E+04	1.06E+04	1.11E+04	0.00E+00	3.22E+03
	**St.Dv**	6.29E+01	1.31E+00	9.37E-01	5.77E+00	2.90E+03	3.10E+03	1.00E+03	0.00E+00	1.02E+03
	**Comp.**	+	+	+	+	-	-	-	+	
**F10**	**Mean**	6.60E+03	3.75E+03	3.34E+03	3.49E+03	7.10E+03	6.09E+03	4.97E+03	1.73E+03	4.90E+03
	**St.Dv**	1.63E+03	2.54E+02	2.94E+02	4.31E+02	5.34E+02	3.55E+02	5.83E+02	9.53E+02	7.58E+02
	**Comp.**	-	+	+	+	-	-	=	+	
**Total**	**w/t/l**	1/1/8	0/2/8	0/1/9	0/1/9	7/2/1	7/1/2	5/3/2	0/1/9	

**Table 11:** CEC-2017 statistical results for 50D, benchmark functions: 11 to 20. There are three possible comparison values: “-” means that our proposed LCGA performs better than the algorithm under consideration; “+” represents the opposite; and “=” indicates that the statistical analysis found no significant evidence to claim neither algorithm was better than its counterpart. On the last row for each column, the comparison value is displayed in terms of win/tie/loss (w/t/l), where win gives the accumulated minus (“-”) highlighted in green, the tie gives the total number of equals (“=”) found, and loss shows the accumulated plus (“+”) counts highlighted in red.

Fx	Stats	SaDE	JADE	SHADE	MVMO	CV1.0	CVnew	CSsin	RB-IPOP-CMA-ES	LCGA
**F11**	**Mean**	1.09E+02	1.36E+02	1.20E+02	4.74E+01	1.66E+02	1.18E+02	1.17E+01	1.83E+02	3.17E+02
	**St.Dv**	3.54E+01	3.39E+01	2.93E+01	8.72E+00	3.38E+01	1.91E+01	2.91E+01	5.20E+01	3.41E+02
	**Comp.**	+	+	+	+	+	+	+	+	
**F12**	**Mean**	1.11E+05	5.14E+03	5.13E+03	1.29E+03	1.00E+10	1.00E+10	1.00E+10	2.44E+06	3.32E+06
	**St.Dv**	6.20E+04	3.32E+03	2.87E+03	2.79E+02	0.00E+00	0.00E+00	0.00E+00	1.74E+07	1.77E+06
	**Comp.**	+	+	+	+	-	-	-	=	
**F13**	**Mean**	1.21E+03	3.03E+02	2.65E+02	4.37E+01	1.00E+10	9.80E+09	1.10E+10	1.65E+03	2.28E+03
	**St.Dv**	1.45E+03	2.69E+02	1.49E+02	1.76E+01	0.00E+00	1.40E+09	0.00E+00	1.15E+03	2.58E+03
	**Comp.**	+	+	+	+	-	-	-	=	
**F14**	**Mean**	2.18E+03	1.05E+04	2.15E+02	4.85E+01	2.05E+02	3.98E+01	2.23E+04	2.42E+02	1.02E+06
	**St.Dv**	2.20E+03	3.11E+04	7.29E+01	1.21E+01	2.13E+01	1.62E+01	1.72E+04	7.07E+01	9.67E+05
	**Comp.**	+	+	+	+	+	+	+	+	
**F15**	**Mean**	3.35E+03	3.49E+02	3.22E+02	4.46E+01	1.37E+09	2.85E+02	1.13E+04	5.29E+02	3.41E+03
	**St.Dv**	2.79E+03	4.42E+02	1.42E+02	1.12E+01	3.47E+09	3.54E+02	6.02E+03	1.15E+02	3.51E+03
	**Comp.**	=	+	+	+	-	+	-	+	
**F16**	**Mean**	8.17E+02	8.56E+02	7.33E+02	8.40E+02	1.53E+03	1.44E+03	7.23E+02	8.90E+02	1.82E+03
	**St.Dv**	2.34E+02	1.75E+02	1.88E+02	1.93E+02	2.74E+02	2.10E+02	1.79E+02	3.66E+02	4.86E+02
	**Comp.**	+	+	+	+	+	+	+	+	
**F17**	**Mean**	5.08E+02	6.00E+02	5.16E+02	5.19E+02	1.25E+03	1.13E+02	1.50E+02	3.98E+02	1.47E+03
	**St.Dv**	1.53E+02	1.21E+02	1.11E+02	1.33E+02	1.85E+02	1.92E+02	1.16E+02	1.58E+02	3.81E+02
	**Comp.**	+	+	+	+	+	+	+	+	
**F18**	**Mean**	3.24E+04	1.89E+02	1.89E+02	4.17E+01	5.21E+02	1.51E+02	1.73E+05	3.57E+02	2.40E+06
	**St.Dv**	1.68E+04	1.25E+02	1.03E+02	1.94E+01	1.19E+02	4.43E+01	7.91E+04	1.56E+02	1.21E+06
	**Comp.**	+	+	+	+	+	+	+	+	
**F19**	**Mean**	1.13E+04	3.24E+02	1.59E+02	1.73E+01	1.73E+02	5.57E+01	5.84E+03	1.39E+02	1.18E+04
	**St.Dv**	1.68E+04	1.25E+03	5.68E+03	5.13E+00	4.17E+02	1.10E+01	3.15E+03	4.77E+01	6.37E+03
	**Comp.**	=	+	+	+	+	+	+	+	
**F20**	**Mean**	3.52E+02	4.38E+02	3.33E+02	3.29E+02	1.05E+03	2.81E+02	2.31E+02	5.47E+02	1.10E+03
	**St.Dv**	1.50E+02	1.33E+02	1.20E+02	1.47E+02	2.14E+02	1.65E+02	9.73E+01	2.33E+02	3.03E+02
	**Comp.**	+	+	+	+	=	+	+	+	
**Total**	**w/t/l**	0/2/8	0/0/10	0/0/10	0/0/10	3/1/6	2/0/8	3/0/7	0/2/8	

**Table 12:** CEC-2017 statistical results for 50D, benchmark functions: 21 to 30. There are three possible comparison values: “-” means that our proposed LCGA performs better than the algorithm under consideration; “+” represents the opposite; and “=” indicates that the statistical analysis found no significant evidence to claim neither algorithm was better than its counterpart. On the last row for each column, the comparison value is displayed in terms of win/tie/loss (w/t/l), where win gives the accumulated minus (“-”) highlighted in green, the tie gives the total number of equals (“=”) found, and loss shows the accumulated plus (“+”) counts highlighted in red.

Fx	Stats	SaDE	JADE	SHADE	MVMO	CV1.0	CVnew	CSsin	RB-IPOP-CMA-ES	LCGA
**F21**	**Mean**	2.87E+02	2.51E+02	2.33E+02	2.77E+02	5.41E+02	1.18E+02	1.57E+02	2.06E+02	3.92E+02
	**St.Dv**	1.36E+01	9.63E+00	5.11E+00	1.60E+01	6.27E+01	8.77E+01	9.74E+01	3.23E+00	3.73E+01
	**Comp.**	+	+	+	+	-	+	+	+	
**F22**	**Mean**	2.92E+03	3.33E+03	3.17E+03	3.26E+03	7.33E+03	5.77E+03	1.00E+02	2.05E+03	5.48E+03
	**St.Dv**	3.24E+03	1.80E+03	1.55E+03	1.71E+03	1.99E+03	3.64E+02	3.91E-01	1.76E+03	1.55E+03
	**Comp.**	+	+	+	+	-	=	+	+	
**F23**	**Mean**	5.22E+02	4.79E+02	4.59E+02	5.04E+02	7.74E+02	1.87E+02	4.51E+02	4.23E+02	7.64E+02
	**St.Dv**	2.05E+01	1.17E+01	8.75E+00	1.71E+03	8.06E+01	5.11E+01	7.88E+01	1.39E+01	8.78E+01
	**Comp.**	+	+	+	=	=	+	+	+	
**F24**	**Mean**	5.89E+02	5.31E+02	5.31E+02	5.83E+02	8.32E+02	3.25E+02	6.87E+02	4.91E+02	1.10E+03
	**St.Dv**	1.86E+01	7.62E+00	7.45E+00	1.69E+01	1.21E+01	8.95E+01	3.57E+01	5.73E+00	1.29E+02
	**Comp.**	+	+	+	+	+	+	+	+	
**F25**	**Mean**	5.71E+02	5.19E+02	5.06E+02	5.09E+02	5.43E+02	4.70E+02	4.26E+02	4.81E+02	5.66E+02
	**St.Dv**	3.05E+01	3.48E+01	3.64E+01	3.12E+01	1.51E+01	2.26E+01	2.08E+01	5.18E+00	2.34E+01
	**Comp.**	=	+	+	+	+	+	+	+	
**F26**	**Mean**	2.52E+03	1.61E+03	1.41E+03	1.93E+03	2.48E+03	1.16E+03	3.00E+02	6.55E+02	4.89E+03
	**St.Dv**	3.37E+02	1.21E+02	9.78E+01	2.86E+02	1.88E+03	1.56E+03	4.57E-02	3.01E+02	9.51E+02
	**Comp.**	+	+	+	+	+	+	+	+	
**F27**	**Mean**	7.10E+02	5.50E+02	5.49E+02	5.43E+02	7.38E+02	4.53E+02	5.97E+02	6.08E+02	5.89E+02
	**St.Dv**	6.65E+01	2.34E+01	2.78E+01	1.75E+01	8.21E+01	7.17E+01	3.22E+01	5.86E+01	1.50E+02
	**Comp.**	-	+	+	+	-	+	=	=	
**F28**	**Mean**	4.99E+02	4.91E+02	4.79E+02	4.64E+02	4.94E+02	4.58E+02	4.13E+02	4.70E+02	5.47E+02
	**St.Dv**	1.53E+01	2.08E+01	2.41E+01	1.50E+01	1.93E+01	2.33E-01	1.83E+01	1.94E+01	3.80E+01
	**Comp.**	+	+	+	+	+	+	+	+	
**F29**	**Mean**	5.11E+02	4.77E+02	4.87E+02	4.89E+02	1.69E+03	1.45E+03	8.03E+02	6.69E+02	1.20E+03
	**St.Dv**	1.37E+02	8.06E+01	1.05E+02	1.40E+01	2.29E+02	1.68E+02	1.24E+02	1.99E+02	3.10E+02
	**Comp.**	+	+	+	+	-	-	+	+	
**F30**	**Mean**	8.07E+05	6.68E+05	6.82E+05	5.81E+05	4.64E+06	6.02E+05	1.64E+05	6.46E+06	3.29E+03
	**St.Dv**	8.33E+04	9.25E+04	8.51E+04	1.02E+04	8.59E+06	2.99E+04	6.25E+05	5.07E+06	3.40E+03
	**Comp.**	-	-	-	-	-	-	-	-	
**Total**	**w/t/l**	2/1/7	1/0/9	1/0/9	1/1/8	5/1/4	2/1/7	1/1/8	1/1/8	

**Table 13:** CEC-2017 summarized statistical results for 50D.

Fx	Stats	SaDE	JADE	SHADE	MVMO	CV1.0	CVnew	CSsin	RB-IPOP-CMA-ES	LCGA
**F1–F10**	**w/t/l**	1/1/8	0/2/8	0/1/9	0/1/9	7/2/1	7/1/2	5/3/2	0/1/9	LCGA
**F11–F20**	**w/t/l**	0/2/8	0/0/10	0/0/10	0/0/10	3/1/6	2/0/8	3/0/7	0/2/8	LCGA
**F21–F30**	**w/t/l**	2/1/7	1/0/9	1/0/9	1/1/8	5/1/4	2/1/7	1/1/8	1/1/8	LCGA
**Total**	**w/t/l**	3/4/23	1/2/27	1/1/28	1/2/27	15/4/11	11/2/17	9/4/17	1/4/25	LCGA

## Data Availability

All the (LCGA code https://github.com/jcarlosfelix/lcga/tree/main/algorithm, accessed on 10 July 2024), (COCO experiment results https://github.com/jcarlosfelix/lcga/tree/main/bench_COCO/LCGA, accessed on 10 July 2024), and (CEC-2017 benchmark statistics https://github.com/jcarlosfelix/lcga/tree/main/bench_CEC2017, accessed on 10 July 2024) are available at the following (GitHub repository https://github.com/jcarlosfelix/lcga, accessed on 10 July 2024), or go to the next url: https://github.com/jcarlosfelix/lcga, accessed on 10 July 2024.

## Abbreviations

The following abbreviations are used in this manuscript:

GA	Genetic Algorithm
PSO	Particle Swarm Optimization
COCO	Comparing Continuous Optimizers
CEC	Congress on Evolutionary Computation
SSGA	Steady-State Genetic Algorithm
ES	Evolution Strategy
EAs	Evolutionary Algorithms
CMA-ES	Covariance Matrix Adaptation Evolution Strategy
LCGA	Life Cycle Genetic Algorithm
EDAs	Estimation of Distribution Algorithms
GWO	Grey Wolf Optimizer
CEC-2017	Congress on Evolutionary Computation 2017
